# Fingerprinting Chemical Markers in the Mediterranean Orange Blossom Honey: UHPLC-HRMS Metabolomics Study Integrating Melissopalynological Analysis, GC-MS and HPLC-PDA-ESI/MS

**DOI:** 10.3390/molecules28093967

**Published:** 2023-05-08

**Authors:** Konstantinos M. Kasiotis, Eirini Baira, Styliani Iosifidou, Electra Manea-Karga, Despina Tsipi, Sofia Gounari, Ioannis Theologidis, Theodora Barmpouni, Pier Paolo Danieli, Filippo Lazzari, Daniele Dipasquale, Sonia Petrarca, Souad Shairra, Naglaa A. Ghazala, Aida A. Abd El-Wahed, Seham M. A. El-Gamal, Kyriaki Machera

**Affiliations:** 1 Laboratory of Pesticides’ Toxicology, Department of Pesticides Control and Phytopharmacy, Benaki Phytopathological Institute, 145 61 Kifissia, Greecei.theologidis@bpi.gr (I.T.);; 2 General Chemical State Laboratory, Independent Public Revenue Authority (A.A.D.E.), 16 An. Tsocha Street, 115 21 Athens, Greece; 3 Laboratory of Apiculture, Institute of Mediterranean & Forest Ecosystems, ELGO DHMHTRA, 115 28 Athens, Greece; 4 Department of Agriculture and Forest Sciences (DAFNE), University of Tuscia, Via. S. Camillo de Lellis snc, 01100 Viterbo, Italy; 5 Consorzio Nazionale Produttori Apistici (CONAPROA), Via N. Guerrizio, 2, 86100 Campobasso, Italy; 6 Biological Control Department, Plant Protection Research Institute, Agricultural Research Center, Giza 12619, Egypt; 7 Department of Bee Research, Plant Protection Research Institute, Agricultural Research Center, Giza 12627, Egypt; 8 Medicinal and Aromatic Plants Research Department, Horticulture Research Institute, Agricultural Research Center, Giza 12619, Egypt

**Keywords:** citrus, honey, aromatic medicinal plants, pollen, metabolomics, mass spectrometry, HRMS, melissopalynology

## Abstract

(1) Background: Citrus honey constitutes a unique monofloral honey characterized by a distinctive aroma and unique taste. The non-targeted chemical analysis can provide pivotal information on chemical markers that differentiate honey based on its geographical and botanical origin. (2) Methods: Within the PRIMA project “PLANT-B”, a metabolomics workflow was established to unveil potential chemical markers of orange blossom honey produced in case study areas of Egypt, Italy, and Greece. In some of these areas, aromatic medicinal plants were cultivated to enhance biodiversity and attract pollinators. The non-targeted chemical analysis and metabolomics were conducted using ultra-high-performance liquid chromatography high-resolution mass spectrometry (UHPLC-HRMS). (3) Results: Forty compounds were disclosed as potential chemical markers, enabling the differentiation of the three orange blossom honeys according to geographical origin. Italian honey showed a preponderance of flavonoids, while in Greek honey, terpenoids and iridoids were more abundant than flavonoids, except for hesperidin. In Egyptian honey, suberic acid and a fatty acid ester derivative emerged as chemical markers. New, for honey, furan derivatives were identified using GC-MS in Greek samples. (4) Conclusions: The application of UHPLC-HRMS metabolomics combined with an elaborate melissopalynological analysis managed to unveil several potential markers of Mediterranean citrus honey potentially associated with citrus crop varieties and the local indigenous flora.

## 1. Introduction

Honey is a naturally sweet food commodity produced by honey bees, used for thousands of years as a sweet, nourishing natural substance or for therapeutic purposes. This is confirmed by historical findings from ancient times that highlight the importance of honey and other bee products for many civilizations (Egyptians, Greeks, Romans, among others) in the prevention and treatment of various diseases [1]. Among the plethora of bioactive compounds that honey contains, organic acids and flavonoids are the driving force behind this activity [2,3]. Honey’s chemical composition varies according to its botanical and geographical origins [4]. In the context of monofloral honeys, citrus honey is a pale-yellow perlaceous honey with a distinctively fresh and floral orange blossom aroma and a dominantly sweet and lasting taste of citrus [5] that renders it among the most sought honeys worldwide [6]. Citrus honey is produced mainly in the Mediterranean basin, which includes European, African, and Asia countries (such as Spain, Italy, Egypt, Turkey, and Greece) that account for 20% of the world’s citrus production [7]. One way to improve the fruit set and productivity of citrus trees (even though it is cultivar-dependent) is to place beehives to pollinate them. Honey bees show a preference for the nectar of citrus flowers and pollen. In this way, a mutual benefit (citrus crops and honey) is achieved, as the specific characteristics of the crop are improved, and excellent honey is produced [8]. 

Honey is much more expensive to produce than sugar syrups, making it the third food product in the world vulnerable to fraudulent practices [9]. Europe has established a legislative framework safeguarding honey quality [10,11]; however, adulterated honey (not corresponding to the quality characteristics of the authentic honey designated in the label) is an ongoing problem in the European market. 

Therefore, the discovery and establishment of robust chemical markers associated with the botanical and geographical origin that will integrate melissopalynological results seem the most realistic and parallel contemporary approach. Physicochemical parameters, such as 5-hydroxymethylfurfural (HMF), diastase number, and electrical conductivity, are also complementary information on the purity of citrus honey [10,12]. From a chemical perspective, volatile (e.g., terpenes) and semi-volatile-aroma compounds are extensively studied by gas chromatography coupled to mass spectrometry (GC-MS) as citrus honey markers [13,14]. Among the volatile compounds, linalool oxide, lilac aldehydes and alcohols [15], and α-4-dimethyl-3-cyclohexene-1-acetaldehyde are reported as the most significant floral markers [16]. Several studies have investigated phenolic compounds in honeys from different citrus species and geographical origins [6]. Among them, the flavonoids usually detected are chrysin, quercetin, hesperetin, hesperidin, pinocembrin, kaempferol, luteolin, rutin, and myricetin. Sinensal isomers and methyl anthralinate at concentrations >0.5 ppm [17,18] are also characteristic markers for citrus honey. Other compounds (such as narirutin, rhoifolin, didymin, etc.) discovered in authenticity studies and present in citrus products can potentially be used [19]. Many studies have also evaluated or reviewed the mineral profile of honeys [20] associated with chemometric analysis to classify citrus honey according to its geographical and/or botanical origin [21]. Though it is evident that much work has been carried out on citrus honey chemical analysis, there is still room for research findings that will improve its recognizability and position in the global market. 

High-resolution mass spectrometry (HRMS) has a decisive role in the investigation of natural products due to its inherent advantage of detecting molecules that fall outside the scope of targeted chemical analytical methods [22,23]. An untargeted HRMS-based metabolomics approach has been applied in the recent past for honey [24] and recently by our group to bee products [22,25]. One of its advantages is that depending on the exact analytical technique used, up to thousands of metabolites can be evaluated, revealing potential biomarkers [26]. Untargeted metabolite profiling has been widely used to identify markers via analysis of a large amount of data from different samples under different conditions using multivariate statistics and library matching [27,28]. Overall, still, unidentified or unexploited markers can be elucidated and offer significant advantages in the classification of honeys and the attribution of unique properties connected with nutritional claims. In this sense, within the PLANT-B project (a project funded by the PRIMA Foundation, European Union), the intervention of aromatic medicinal plants (AΜPs), as designed, is of high importance since the AMPs can be significant nutritional sources for bees, while some of them can supply high-quality honey with constituents bearing medicinal properties. More specifically, based on AMPs cultivated either within crops or in the field margins, the literature has several examples of inherent compounds or secondary metabolites that are transferred from plants to honey and function as markers of origin [29]. 

More specifically, the PLANT-B metabolomics approach was built upon the non-targeted chemical analysis conducted using ultra-high-performance liquid chromatography high-resolution mass spectrometry UHPLC-HRMS (Q-Exactive Orbitrap platform). This approach is subsidized by the use of large databases for chemical identification covering an extensive list of phytochemicals, including flavonoids, flavones, flavanones, and alkaloids, that will potentially unveil new markers [30] or verify existing ones in complementary mode with a targeted LC-PDA-ESI/MS approach. The use of classical GC-MS to elucidate the prevalence of GC amenable compounds (volatile and semi-volatile compounds, such as terpenoids) implicated in the discrimination of botanical origin is also incorporated in the chemical scrutiny. For this task, a sufficient number of honey samples were collected and processed from each production area for the first year of harvest (e.g., citrus and/or multifloral honey) in order to acquire analytical data. AMPs cultivated in the case study fields during the first year of the project PLANT-B is expected to function as supplementary nectar and pollen sources to the bees and potentially contribute to the chemical arsenal of citrus honey, along with the local indigenous flora. 

Hence, the primary goal of this work was to explore how untargeted metabolomics via a UHPLC-HRMS platform would benefit the discovery of newly reported compounds and chemical markers in citrus honey from three distinct Mediterranean countries, thus assessing the differentiation among the case study regions. This effort was substantiated by a detailed melissopalynological analysis, complementary GC-MS, and targeted HPLC-PDA-ESI/MS analysis contributing to the identification of new GC amenable constituents and quantification of LC amenable compounds. 

## 2. Results and Discussion

### 2.1. Chemometrics

Honey samples originated in most cases from citrus (orange) orchards. In some of the orchards, AMPs were installed (the average range of AMPs/total surface of the citrus orchards under study in Sicily was equal to 117 m^2^, in Greece, it reached 80 m^2^, while in Egypt, it averaged 1820 m^2^), yet their effect on honey composition was questionable due to incomplete plant growth in the first year of the program. Nevertheless, all plants in the surrounding areas were recorded, and melissopalynological analysis has unveiled the potential floral sources. 

**Figure 1:**
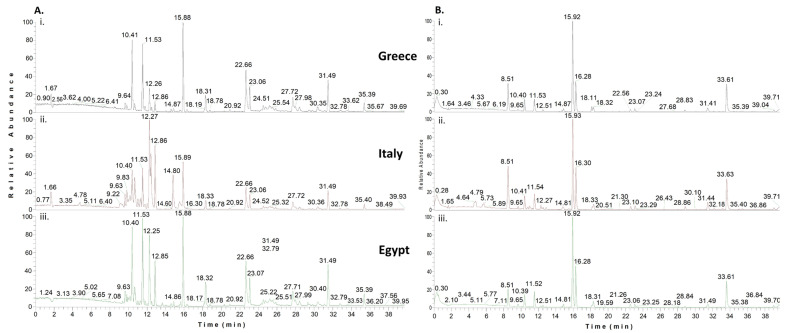
Representative chromatograms of citrus honey extracts from Greece (i), Italy (ii), and Egypt (iii) after UHPLC-HRMS analyses in negative (**A**) and positive (**B**) ionization modes.

**Figure 2:**
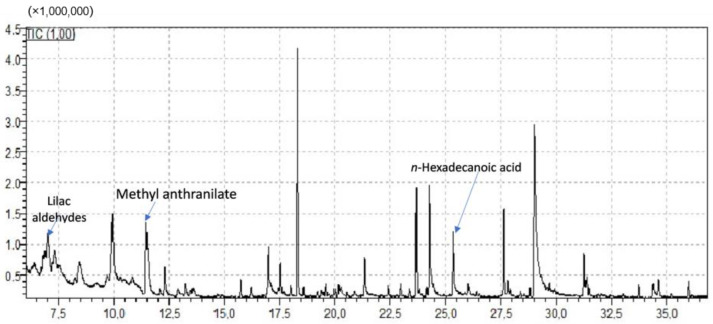
HS-SPME GC-MS full scan chromatogram of a Greek citrus honey extract (selected chemicals’ peaks pointed out).

Despite the domination of citrus nectar (in the studied honey samples), the HRMS analysis and subsequent chemometrics managed to disclose compounds differentially increased among the three countries. These chemical markers are presented in Table 1 and emphasize the importance of both geographical and botanical origin. Some representative chromatograms from UHPLC-HRMS and GC-MS analysis are shown in Figure 1 and Figure 2, respectively. Valuable chemometric approaches, including citrus honey as well, have also been presented in the past, incorporating physicochemical traits [31], and currently, focusing on volatile compounds of honeys (volatilome) [32].

**Figure 3:**
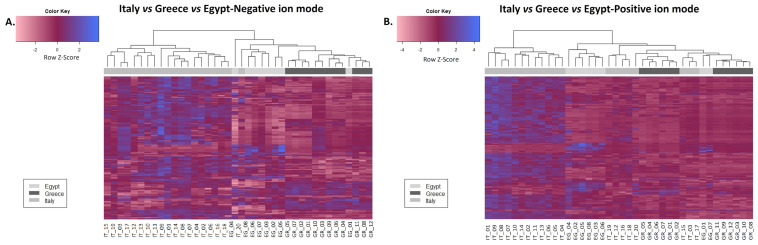
Heatmap analysis in honey extracts from Greece, Italy, and Egypt derived from UHPLC-HRMS analysis in the negative (**A**) and positive (**B**) ion mode. The heatmaps indicate the relative content of secondary metabolites and compounds among the different countries.

In order to investigate the differences between the honey extracts, taking into account their geographical origin, a statistical analysis was performed based on the results from the UHPLC-HRMS analyses. Firstly, a heatmap was generated for both negative and positive modes to investigate the relative content of secondary metabolites and compounds among the different countries. As shown in Figure 3, citrus honey from Italy exhibited more differentially increased compounds in the comparisons (between countries).

**Figure 4:**
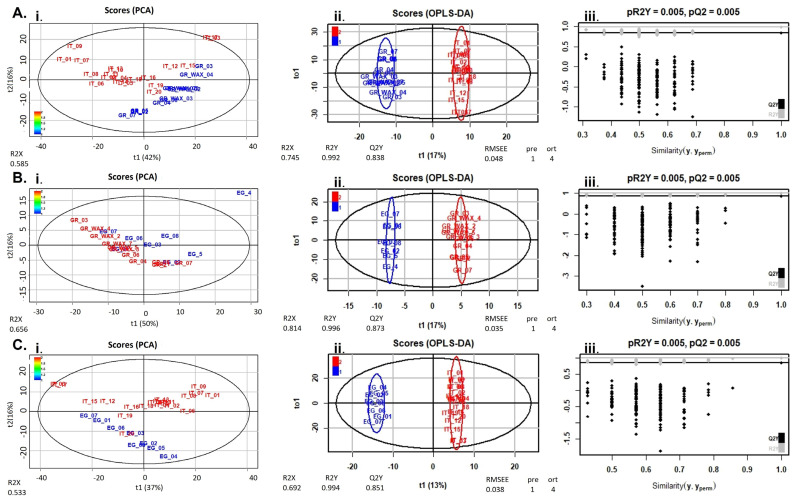
PCA (**Ai**,**Bi**,**Ci**), OPLS-DA (**Aii**,**Bii**,**Cii**) score plots and permutation tests (**Aiii**,**Biii**,**Ciii**) after the comparison of honey extracts from Greece (blue) and Italy (red) (**A**), Greece (red) and Egypt (blue) (**B**), and Italy (red) and Egypt (blue) (**C**), in the negative ion mode. The goodness of fit and prediction of the models were for PCA analysis (**Ai**) R2X = 0.585, (**Bi**) R2X = 0.656, (**Ci**) R2X = 0.533 and for OPLS-DA analysis (**Aii**) R2X = 0.745, R2Y = 0.992, Q2Y = 0.838, (**Bii**) R2X = 0.814, R2Y = 0.996, Q2Y = 0.873, (**Cii**) R2X = 0.692, R2Y = 0.994, Q2Y = 0.851.

**Figure 5:**
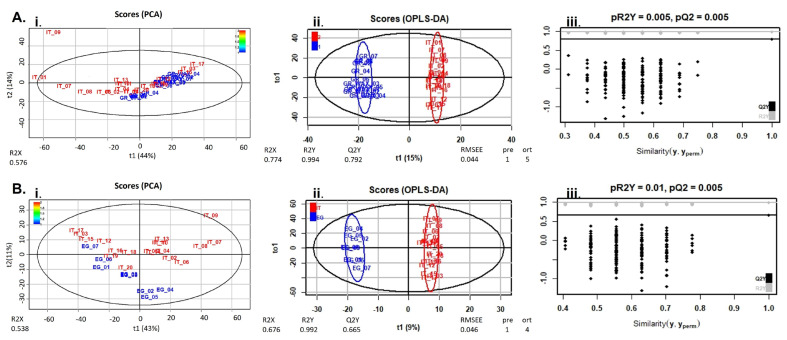
PCA (**Ai**,**Bi**), OPLS-DA (**Aii**,**Bii**) score plots and permutation tests (**Aiii**,**Biii**) after the comparison of honey extracts from Greece (blue) and Italy (red) (**A**) and Italy (red) and Egypt (blue) (**Β**), in the positive ion mode. The goodness of fit and prediction of the models were for PCA analysis (**Ai**) R2X = 0.576, (**Bi**) R2X = 0.538 and for OPLS-DA analysis (**Aii**) R2X = 0.774, R2Y = 0.994, Q2Y = 0.792, (**Bii**) R2X = 0.676, R2Y = 0.992, Q2Y = 0.665.

In this context, PCA and OPLS-DA were calculated using R for visualization of any metabolic clustering of the different groups of samples. As presented in Figure 4 and Figure 5, statistical analysis managed to separate honey samples when these were compared bilaterally (country level), in the negative mode for all countries (Figure 4) and for Greece vs. Italy and Italy vs. Egypt, in the positive ion mode (Figure 5). The statistical analysis revealed several compounds to be differentially increased among the three countries. These chemical markers are presented in Table 1 and emphasize the importance of both geographical and botanical origin, also considering other honeybee foraging plants apart from installed AMPs. It is important to mention that the integration of the four non-citrus Egyptian honey samples in the metabolomics provided comparable findings when solely citrus honey was considered. Consequently, they were not excluded, and the totality of samples is presented. In the same context, one honey obtained from the Italian market (sample number 20) did not correspond to its label as citrus honey, verified both by HRMS and melissopalynological analysis.

**Table 1:** Main compounds differentially increased in the honey samples from the three countries *.

Compound Annotation	Monoisotopic Mass (Da) Experimental_tR (min)	Molecular Formula	Italy	Greece	Egypt	Compound Class	Adduct Ion	MS/MS Fragment Ions (*m*/*z*)
4-Hydroxycinnamyl aldehyde	148.0514_9.06	C_9_H_8_O_2_	+++	d ^a^	d	Organic acid	[M-H]^−^	119.05/101.04
3-Phenyllactid acid	166.0621_10.12	C_9_H_10_O_3_	+++	d	++(Gr)	Organic acid	[M-H]^−^	147.04/119.05
Ferulic acid	194.0572_9.30	C_10_H_10_O_4_	+++	d	d	Organic acid	[M-H]^−^	134.03/178.02 **
Lumichrome	242.0802_10.86	C_12_H_10_N_4_O_2_	+++	d	d	Riboflavin metabolite	[M-H]^−^	198.07/106.02
Formononetin	268.0737_14.15	C_16_H_12_O_4_	+++	d	d	Isoflavone	[M-H]^−^	252.04
Glycitein	284.0686_16.43	C_16_H_12_O_5_	+++	d	d	Isoflavone	[M-H]^−^	268.03
Hispidulin	300.0635_13.54	C_16_H_12_O_6_	+++	d	d	Flavone	[M-H]^−^	284.03, 136.98 **
3,7-Di-*O*-methyl quercetin	330.0742_13.92	C_17_H_14_O_7_	+++	d	d	Flavanol	[M-H]^−^	271.02/299.02/314.04
Naringenin	272.0685_12.49	C_15_H_12_O_5_	+++	d	d	Flavanone	[M-H]^−^	151.00/119.05 **
Sakuranetin	286.0842_14.79	C_16_H_14_O_5_	+++	d	d	Flavanone	[M-H]^−^	119.04/165.01 **
Dihydrokaempferol (Aromadendrin)	288.0634_9.73	C_15_H_12_O_6_	+++	d	d	Flavanonol	[M-H]^−^	125.02/259.06
Chrysin	254.0578_15.57	C_15_H_10_O_4_	+++	d	d	Flavone	[M-H]^−^	253.05/143.05 **
N-Feruloyltyramine	313.1315_14.40	C_18_H_19_NO_4_	+++	d	d	Amide	[M+H]^+^	120.08/103.05
Secologanate	374.1214_6.59	C_16_H_22_O_10_	+++	d	d	Terpenoid	[M-H]^−^	165.05/147.04
Cinncassiol B	422.1919_12.33	C_20_H_32_O_8_	+++	d	d	Terpenoid	[M+Na-2H]^−^	153.09
Abscisic acid	264.1362_11.07	C_15_H_20_O_4_	++(Eg)	d	d	Sesquiterpene	[M-H]^−^	204.11/219.13 **
Corchorifatty acid F	328.2253_14.51	C_18_H_32_O_5_	+++	d	d	Fatty acid	[M-H]^−^	211.13/229.14
Isorhamnetin 3-rutinoside 4”-rhamnoside	770.2267_10.55	C_34_H_42_O_20_	+++	d	d	Flavonoid glycoside	[M+H]^+^	301.07/286.04
Pinocembrin	256.0735_14.90	C_15_H_12_O_4_	+++	++(Eg)	d	Flavanone	[M+H]^+^	153.01/131.04 **
Hesperetin	302.0790_12.71	C_16_H_14_O_6_	+++	d	d	Flavanone	[M+H]^+^	153.01/134.03 **
Rutin	610.1537_9.23	C_27_H_30_O_16_	+++	d	d	Flavone	[M+H]^+^	287.05/317.06 **
Sebacic acid	202.1206_11.41	C_10_H_18_O_4_	++(GR)	d	d	Carboxylic acid	[M+H]^+^	121.10/97.06
Scopoletin	192.0424_8.21	C_10_H_8_O_4_	++(GR)	d	d	Coumarin	[M+H]^+^	193.04/133.02 **
L-phenylalanine	165.0790_2.93	C_9_H_11_NO_2_	++(GR)	d	d	Amino acid	[M+H]^+^	120.08/103.05
Acetophenone	120.0576_10.81	C_8_H_8_O	++(GR)	d	d	Aromatic ketone	[M+H]^+^	121.06/103.05 **
4-Hydroxyquinoline	145.0527_4.78	C_9_H_7_NO	++(GR)	d	d	Quinoline	[M+H]^+^	128.04
3Z-Hexenyl 2R-hydroxy-3-methylbutyrate	200.1414_13.07	C_11_H_20_O_3_	d	++(It)	d	Fatty acyls/Fatty esters	[M+H]^+^	67.05/55.05
(3S, 7R)-iso-jasmonic acid	210.1251_13.11	C_12_H_18_O_3_	d	++(It)	d	Fatty acid	[M-H]^−^	93.06/81.06
14-hydroxy-12-tetradecenoic acid	242.1882_30.19	C_14_H_26_O_3_	d	++(It)	d	Fatty acid	[M+H]^+^	95.08/81.06
9Z-Hexadecenamide	253.2405_25.59	C_16_H_31_NO	d	++(It)	d	Fatty amide	[M+H]^+^	69.06/83.08
Hesperidin	610.1903_10.14	C_28_H_34_O_15_	d	++(It)	d	Flavanone glycoside	[M-H]^−^	301.07/151.00 **
10-Hydroxy-2-decenoic acid	186.1248_13.04	C_10_H_18_O_3_	d	++(It)	d	Fatty acid	[M-H]^−^	185.11/139.11 **
Hallactone B	440.1160_9.69	C_20_H_24_O_9_S	d	+++	d	Terpenoid	[M-H]^−^	393.10
Provincialin	518.2153_12.68	C_27_H_34_O_10_	d	+++	d	Terpenoid	[M-H]^−^	111.04
Nepetaside	346.1629_11.87	C_16_H_26_O_8_	d	++(It)	d	Iridoid glucoside	[M-H]^−^	183.10/185.11
Patrinoside	508.2155_10.37	C_21_H_34_O_11_	d	++(It)	d	Iridoid glucoside	[M+FA-H]^−^	183.10/139.11
(4E, 6E, d14:2) sphingosine	241.2041_30.19	C_14_H_27_NO_2_	d	++(It)	d	Amino alcohol	[M+H]^+^	109.10/95.08
Quercetin 3-*O*-sophoroside	626.1491_8.15	C_27_H_30_O_17_	d	++(It)	d	Flavonoid glycoside	[M-H]^−^	300.02/271.02 **
11Z, 13E, 15-Hexadecatrienyl acetate	278.2245_27.67	C_18_H_30_O_2_	d	d	+++	Fatty acid ester	[M-H]^−^	59.01
Suberic acid	174.0884_9.46	C_8_H_14_O_4_	d	d	++(Gr)	Dicarboxylic acid	[M-H]^−^	111.08/173.08 **

*+++ signify relative increase compared to the rest of the two countries based on Log2 fold change, ++ signify relative increase compared to the country in parenthesis based on Log2 fold change, ** verified with analytical standard injections, ^a^: d = detected.

As presented in Table 1, the honey samples from Italy compared to Greece and Egypt were differentially increased in glycerophospholipids, flavonoids, and some terpenic molecules. In the same context, in Egypt (when matched to Greece) fatty acyl glycosides of mono- and disaccharides and carboxylic acids were found to increase. Greece compared to Italy showed increased levels of selected terpenoids, iridoid glycosides, and fatty acids. These differences, presented in detail below, are potentially attributed to the different orange tree varieties used in the case studies of the project, especially between Greece and Italy, where the majority of citrus-based honey came from, and the geographical origin. A recent study showed the effect of geographical origin on the differences concerning the chemical composition among the same types of honey [33]. In another domain, recent work on the chemical composition of juices originating from several European pear cultivars has shown that such differences are cultivar-dependent [34].

#### 2.1.1. Flavonoids

The use of flavonoids as chemical markers of honey’s botanical origin has been reported by several research groups [35,36]. Similar patterns have been used to identify bee pollen’s respective origin [37]. In this work, attention was given to discriminate citrus honey from three distinct Mediterranean countries. This task was challenging since the majority of samples were of citrus crop origin and the margins of differentiation among them, at first reading, seemed limited. 

**Figure 6:**
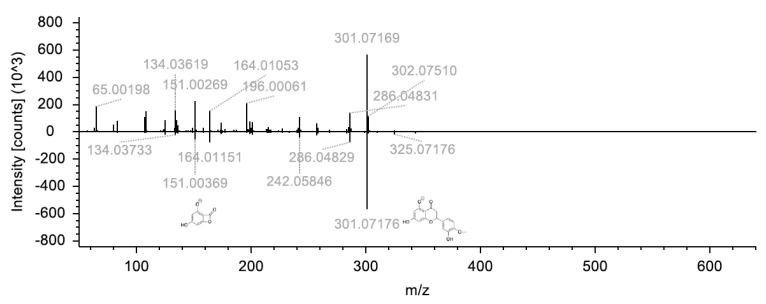
Hesperidin annotation based on MS/MS fragmentation pattern using mzCloud database. The top panel is the MS/MS pattern of the raw file from a Greek citrus honey sample, whereas the bottom panel is the MS/MS pattern of Hesperidin from the mzCloud database.

Hesperidin (flavanone) and its aglycone form, hesperetin, are characteristic flavonoids of citrus honey. Hence, insight into the variability of concentrations among the same type of honey is of the utmost importance. The latter becomes even more significant when attempts are made to discriminate honeys based on geographical criteria. It is noteworthy that hesperetin (tR = 12.71, with 302.0790 *m*/*z* Table 1) was increased in Italian honeys, while hesperidin (tR = 10.14 with 610.1903 *m*/*z* Table 1), a glycoside of hesperetin, was comparatively increased in Greek honeys (Figure 6). Both molecules can be further investigated under a biosynthetic framework. Flavonoids’ biosynthesis has been studied by many research groups, though for several of its members (i.e., hesperetin), it is still unknown. Hence, any observation of their formation and the conditions that govern them is significant. 

It is reported that under specific conditions the content of flavanone glycosides and their aglycones undergoes drastic changes, in order to protect plants against pathogens [38]. In the specific case, since hesperetin is a precursor of hesperidin, it seems that in the specific Greek honey samples, this conversion was favored, proceeding via the formation of the intermediate hesperetin-7-O-β-D-glucoside. Hence, the observed relative difference of hesperetin levels can be attributed to such factors. To strengthen the HRMS results, the targeted HPLC-PDA-ESI/MS chemical analysis verified the differential increase in these two molecules. More specifically, hesperetin levels in Greek citrus honey showed a mean concentration of 0.19 ± 0.04 μg/g honey, while in Italian citrus honey the respective value was 0.49 ± 0.20 μg/g honey (Table S1). On the contrary, for hesperidin, the mean concentration in Greek samples was 3.78 ± 0.51 μg/g honey, while in Italian samples was 1.12 ± 0.16 μg/g honey. In the three Egyptian citrus honey samples, hesperetin and hesperidin mean levels were 0.14 ± 0.04 and 0.71 ± 0.17 μg/g honey, respectively (Table S1). Chrysin (tR = 15.57, with 254.0578 *m*/*z* Table 1), sakuranetin (tR= 14.79, with 286.0842 *m*/*z* Table 1), naringenin (tR = 12.49, with 272.0685 *m*/*z* Table 1), rutin (tR = 9.23, with 610.1537 *m*/*z* Table 1) and pinocembrin (tR = 14.90, with 256.0735 *m*/*z* Table 1) were similarly elevated in Italian citrus honey (mean at 1.24 ± 0.18, 0.09 ± 0.03, 4.26 ± 0.14, 5.81 ± 0.35 and 3.38 ± 0.41 μg/g, respectively), and lesser represented in Greek and Egyptian honey (0.98 ± 0.11, 0.05 ± 0.02, 2.96 ± 0.24, 4.21 ± 0.51 and 2.63 ± 0.27 μg/g and 0.88 ± 0.09, 0.04 ± 0.01, 0.97 ± 0.06, 3.72 ± 0.41 and 2.09 ± 0.31 μg/g, respectively, Table S1). These concentration differences might also reflect strictly regional and varietal characteristics that should not be foreseen as a generic remark for the specific type of honey (of the country of origin). The comparison with literature reports on orange blossom honey showed that the determined levels for the majority of flavonoids are in the same order of magnitude [39,40], though certain differences are observed. More specifically, naringenin’s and rutin’s mean concentrations in Italian and Greek honey (for rutin in Egyptian honey as well) are higher than the ones reported in Spanish honey [39], though in the specific study, only one commercial orange blossom honey sample was analyzed. 

Dihydrokaempferol (tR = 9.73, with 288.0634 *m*/*z* Table 1) increased in Italian honey is an important finding considering that its only report in honey is on stingless bee honey in the form of aromadendrin (its trans isomer) [41]. Hence, it can be further studied as a putative biomarker of Italian citrus honey. Plant sources for this compound mentioned in the literature belong to the families of Cactaceae and Aizoaceae [42]. The Cactacae family is also reported as flora of the case studies in Italy, flowering from April to July (see Tables S4 and S5A,B). Quercetin 3-O-sophoroside (tR = 8.15, with 626.1491 *m*/*z* Table 1) increased in Greek samples has been found at significant amounts in rapeseed honey [40] and trace levels in Diplotaxis honey [43]. Brassicaceae family of plants, from which rapeseed honey is produced is also reported in the Greek case study fields (Brassica/Sinapis, and Draba/Eruca, see also the melissopalynological analysis section and the respective Table). The same compound was also identified in Lithuanian bee pollen [44]. However, until the present work, no connection was made to citrus honey. Therefore, its use as a chemical marker for Greek citrus honey can be further studied. 

Flavonoids not previously reported in citrus honey are also an intriguing task. 3,7-Di-O-methyl quercetin (tR = 13.92, with 330.0742 *m*/*z* Table 1) is a methylated flavanol and polyphenolic metabolite of Rhamnus disperma [45] (Rhamnus genus was identified via melissopalynological analysis in Italian samples, less in Greek and Egyptian samples) also reported as a constituent of Loranthus globosus, exhibiting anti-Alzheimer activity [46]. A rare flavonoid, glycoside isorhamnetin 3-rutinoside 4″-rhamnoside (tR = 10.55, with 770.2267 *m*/*z* Table 1), was putatively annotated and differentially increased in Italian citrus honey. This chemical was reported as a component of the flowers of Cucurbita pepo [47]. In general, Cucurbitaceae (pollen grains identified both in Italian and Greek honey) are plants visited by bees [48], and their fruit set is dependent on pollination [49]; hence, such chemicals are expected in apiculture commodities. Hispidulin (tR = 13.54, with 300.0635 *m*/*z* Table 1, see structure in Figure 6) is a bioactive [50] methylated-oxygenated flavone-documented component of tropical propolis [51]. To our knowledge, it is its first report on honey and apiculture commodities of non-tropical origin. Concerning other common flavonoids among the countries, their concentrations are presented in Table S1.

#### 2.1.2. Fatty and Organic Acids 

Interestingly, in Greek honey samples, the saturated fatty acid, 10-hydroxy-2-decenoic acid (10-HDA) (tR = 13.04, with 186.1248 *m*/*z* Table 1), a known bioactive molecule [52] was distinctively increased. More specifically, 10-HDA is the driving force behind the pronounced biological activity of royal jelly that, to an extent, justifies its increased demand from consumers. The biosynthesis of 10-HDA has gathered large attention from the scientific community. Brown and colleagues suggested sucrose as the precursor of 10-HDA after its administration in worker bees and subsequent monitoring of such fatty acids [53]. Plettner and coworkers moved forward, suggesting a de novo fatty acids synthesis with stearic acid being the precursor [54]. 

The differentiation in the levels of 10-HDA is an intriguing issue not only for this molecule but also for all compounds annotated in this study. Another reading for some of the constituents that are found in other apiculture matrices (such as propolis) than honey is that these bioactive compounds are transferred to honey at the onset of nectar’s deposition in the honeycomb [43]. Another hydroxydecenoic acid counterpart, putatively annotated in citrus honey of the present work, is 14-hydroxy-12-tetradecenoic acid (tR = 30.19, with 242.1882 *m*/*z* Table 1). Considering the already acknowledged bioactivity of 10-HDA, the disclosure of this molecule can be an added value for Mediterranean citrus honey. (3S, 7R)-iso-jasmonic acid (tR = 13.11, with 210.1251 *m*/*z* Table 1) was differentially increased in Greek citrus honey. The latter is a signal molecule involved in the octadecanoic pathway, produced in plants, especially after insects’ attacks [55]. In this context, jasmonic acid is a phytohormone reported in monofloral raw honeys [22,56], which along with other jasmonates, demonstrate anticancer activities [57]. Phytohormones are found in plants’ nectar [58] and, after foraging, can be transferred inside the beehives. Ferulic acid (tR = 9.30, with 194.0572 *m*/*z*, Table 1) is a known component of honey and apiculture matrices, verified in this study. Its quantitative determination showed higher levels in Italian honey (mean levels at 1.22 ± 0.28 μg/g) compared to Greek and Egyptian ones (0.96 ± 0.21 μg/g and 0.65 ± 0.13 μg/g, respectively). 

Corchorifatty acid F (tR = 14.51, with 328.2253 *m*/*z* Table 1) is a challenging molecule when viewed from the perspective of the components of honey. More specifically, this acid was reported as a constituent of the leaves of *Corchorus olitorius* L. (Tiliaceae) [59], and of *Coryphantha macromeris* (Cactaceae) [60] native in the United States of America and Mexico. Corchorifatty acid F connects with the plant *Euphorbia hirta* L. The latter is native to tropical and subtropical regions of Central South America, Asia, and Africa. Nevertheless, in the Mediterranean region, plants of both the Euphorbiaceae and Cactaceae families exist and recognized in the specific study (see Tables S4 and S5A,B); therefore, its putative annotation can be attributed to such plants. Last but not least, another corchorifatty acid (B) was identified in the Solanaceae family and *Solanum americanum* Mill., in particular [61], a family of plants abundant in Europe as well. 

#### 2.1.3. Terpenes 

**Figure 7:**
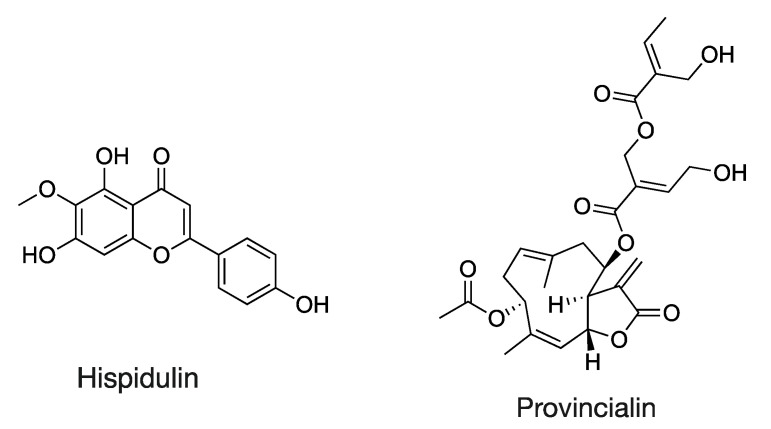
Indicative chemical markers characteristic of Mediterranean citrus honey.

All Greek and some of the Italian honeys displayed several terpenoids in their chemical arsenal. More specifically, the qualitative analysis and semi-quantitative approach based on the relative abundances obtained (peak areas) for Greek citrus honey showed a constant representation of certain terpenoids. The latter might be attributed to the “homogeneity” and the non-scattered profile of the Greek case study fields, which are all located within an overall perimeter of 15 km. Among the compounds annotated are hallactone B (tR = 9.69, with 440.1160 *m*/*z* Table 1) and provincialin (tR = 12.68, with 518.2153 *m*/*z* Table 1, see structure in Figure 7). These terpenoids belong to the class of limonoids, which are highly oxygenated triterpenic molecules distributed in several plants’ families, exemplified by the citrus crops and plants of the Cucurbitaceae families. To our knowledge for provincialin and hallactone B, it is their first report in honey. Elaborating on terpenes, it is worth noting that different terpenes were found to be elevated in Italian honey. The latter can be a point to distinct orange honey among regions and countries. However, additional sampling and data are needed to corroborate such conclusions since it is impossible to “avoid” chemical substances derived from other plants. A rare terpenic molecule among them is secologanate (tR = 6.59, with 374.1214 *m*/*z* Table 1). This compound reference is focused on the *Dendrobium officinale,* which is a special orchid species native to Asia [62]. Nevertheless, since *Dendrobium* species are cultivated in Europe, used as ornamental plants, and visited by bees, it is possible to identify them as potential sources of secologanate. Cincassiol B (tR = 12.33, with 422.1919 *m*/*z* Table 1), a diterpenic glycoside, has been reported as a constituent in *Descurainia sophia* seeds extract [63]. It is native to Eurasia (including Italy) but, to our knowledge, has never been reported as a honey component. 

Largely different levels of the same compounds or substantially different compositions of terpenoids among crops or plants of the same type have been reported by several groups. Indicatively, terpenoids showed dissimilar contents among different types of tangerines [64]. In another study, the terpenoid variations were also correlated to genotypic differences [65]. Consequently, the results of the differential expression of terpenoids in the present study are not unexpected. 

#### 2.1.4. Other Chemicals 

Lumichrome (tR = 10.86, with 242.0802 *m*/*z* Table 1) is the enzymatic cleavage product of riboflavin. In this context, its annotation in the present work comes to add another evidence to its reference to monofloral honeys [66]. The same group used this molecule as a biomarker of thistle honey [67]. Though formononetin (tR = 14.15, with 268.0737 *m*/*z* Table 1) has been ascribed to acacia, vitex, and linden honey from China [62], this report is its first appearance in citrus honey. Formononetin has also been reported as a constituent of Brazilian red propolis [68], a natural bee product known for its antibacterial, antioxidant, and cytotoxic activities. A precursor of sphingolipids known as parts of biological membranes, sphingosine, was also identified in the present study. The major phospholipid of the honeybee is sphingomyelin, reported since the early 1970s [69]. Greek honey differentially increased (4E, 6E, d14:2) sphingosine (or tetradecasphinga-4E,6E-dienine), (tR = 30.19, with 241.2041 *m*/*z* Table 1)), an amino alcohol first reported in citrus honey in this study. 

4-Hydroxycinnamyl aldehyde (tR = 9.06, with 148.0514 *m*/*z* Table 1) was differentially increased in Italian citrus honey. The latter is a cinnamaldehyde derivative isolated from the rhizomes *Alpinia galanga* (Linn) [70] and in *Salix* species [71]. *Salix alba* L. was reported in the flora of the Italian fields; hence, it can be the potential source of this molecule, though not. It is noteworthy that it is a bioactive molecule inducing the apoptosis of human leukemic cells [72]. Scopoletin (tR = 8.09, with 192.0424 *m*/*z* Table 1), which is reported as a constituent of Greek cotton honey [73], has not been evidenced in citrus honey until the specific report. In the same context, cotton cultivation is not registered in the Greek case study areas to imply the uptake of this component from such an antagonizing crop. However, it is found in Asteraceae, Convolvulaceae, Rubiaceae, Solanaceae, and Moraceae plants (see [74] and references therein), with Asteraceae, Convolvulaceae, and Moraceae, verified in this work. Other components, such as sebacic acid (tR = 11.31, with 202.1206 *m*/*z* Table 1), are fermentation products of the bee gut microbiota [75]. Acetophenone (tR = 10.81, with 120.0576 *m*/*z* Table 1) belongs to the volatile markers of several types of honey, such as chestnut [76], thyme [77], raspberry, heather, and rape honey [78]. Consequently, its relatively high abundance in Italian citrus honey is worth pointing out, though further use as a chemical marker seems limited. Abscisic acid (tR = 11.07, with 264.1362 *m*/*z* Table 1) was relatively increased in Italian citrus honey, exhibiting a mean concentration of 10.01 ± 0.45 μg/g honey, compared to 8.78 ± 0.39 μg/g and 6.86 ± 0.49 μg/g in Greek and Egyptian honey correspondingly. The latter is a known plant hormone and chemical marker of heather honey [22,79]. 

4-Hydroxyquinoline (tR = 4.78, with 145.0527 *m*/*z* Table 1) increased in Greek honey samples expands the heterocyclic portfolio of citrus honey. Quinolines are bioactive components (especially their fused alkaloid structures) rarely reported in honey [80]. However, it is imperative to mention that the specific compound was used as a chemical marker of jujube honey [81]. In addition, it is reported as a constituent of Rutaceae plants, abundant in Greek case study areas.

In the same framework, iridoid components identified increased in the Greek citrus honey are reported for the first time in the present study. Iridoids are glycosides found in various plants, and they bind to glucose. They have the general form of cyclopentopyran and a molecular structure related to iridodial. Nepetaside (tR = 11.87, with 346.1629 *m*/*z* Table 1) has been reported as a constituent of Gentiana [82] and Nepeta species [83]. Both these plant species are reported in Greece [84]. Recently an RP-HPLC-MS study unveiled nepetaside as a secondary metabolite of *Heliotropium crispum* Desf. [85], which is a widespread genus of the Boraginaceae plant family in the Mediterranean region. Therefore, the occurrence of this rare iridoid can be attributed to plants of this family that exhibited high frequency in Greek honey samples. For patrinoside (tR = 10.37, with 508.2155 *m*/*z* Table 1), another iridoid glycoside, a known component of Valeriana species [86], the picture is clearer. Hence, its annotation in honey samples from Greece can be substantiated based on the occurrence of plant species such as *Sambucus ebulus* L. (Adoxaceae, also found in forestall areas along with fir tree) and *Centranthus* sp. (abundant in the Argolis region, based also on optical observation) that contain this component and its aglycone forms [87,88,89]. Other plants that contain this molecule [90,91] are not reported in the Greek flora. Like terpenes, a different iridoid was distinctively increased in Italian honey than the Greek ones. The detection of iridoids, an underexplored chemical family in honey, is of utmost importance, considering their biological properties and the added value that they induce in the natural products containing them [92]. Other compounds associated with citrus honey, flowers, and nectar or citrus juices [93], such as caffeine [94], exemplified by synephrine (a biogenic amine) also used as a selective marker of citrus honey [95], were not detected in the presented work via LC-HRMS, despite their inclusion in the HPLC-PDA-ESI/MS targeted analytical method. Consequently, the presented work substantiates the complexity of secondary metabolite generation among the same matrices and their use as exclusive chemical markers. 

The majority of plant species reported in this work as potential sources of chemicals annotated, are visited, and pollinated by bees for nectar and pollen collection, therefore the occurrence of these plants’ components in bee products is logical. To substantiate this statement, Gao and colleagues investigated the bioactive arsenal of citrus nectar and honey [96]. Compounds detected in nectar, such as hesperetin, rutin and gallic acid (see Table 1 and Table S1) were also identified in the present study corroborating a high likelihood of their transfer from citrus nectar to honey. Last but not least, flower-plant traits are associated with the motifs of bees’ visits and plant–pollinator interactions [97,98]. Specifically, the variations and frequency of bees’ visitation (and population consequently) in the diversity of flowers that come across during foraging, can affect the degree of chemicals transferred to honey. Hence, it is an additional factor shaping honey’s chemical profile, apart from nectar and pollen composition.

### 2.2. GC-MS Findings

GC-MS analytical results (no full data shown) were in line with the literature findings on citrus honey volatile components. More specifically, methyl anthranilate, lilac aldehydes (all isomers I–IV), and 2,6-dimethyl-2,7-octadiene-1,6-diol, all characteristic markers of citrus honeys [13,14] were identified in this study as well. Similarly, a plethora of saturated fatty acids and common constituents of citrus honey was identified, such as dodecanoic, decanoic, and undecanoic acid, and other aldehydes such as hexadecanal and heptadecanal. Linalool was also putatively identified in some honey samples. Nevertheless, some volatile chemicals firstly reported in citrus honey are worth mentioning. More specifically, and to our knowledge, 5-dodecyldihydro-(3H)-furanone and 5-tetradecyldihydro-2(3H)-furanone (furan derivatives) were identified for the first time in Greek citrus honeys. Dihydrofuranones are reported in several types of honey [76], but not the aforementioned. 

### 2.3. Melissopalynological Analysis 

The relative level of abundance and relative frequency of citrus pollen in unifloral citrus honeys classifies it as under-represented pollen [12,99]. This means that a relatively small percentage of citrus pollen grains is expected in the melissopalynological analysis, with the remainder of pollen representing various plants of the local flora. The legal limits for the relative frequency of citrus pollen in the monofloral citrus honey range between 3% and 20%, regarding the national legislation of several European countries [100].

#### 2.3.1. Melissopalynological Analysis of Citrus Honey from Sicily, Italy

In citrus honey from Sicily (n = 20), 56 plant families were identified (33 of nectariferous and 23 of nectarless plants, Table S5A). The families of which the sum of pollen accounted for 95–100% of the whole pollen spectra of nectariferous plants are shown in Table 2. The Rutaceae family (*Citrus*) was found, as expected, in low relative frequencies (m.v. 6%, range: <1–31%, Table 2). The dominant family was the Fabaceae, present in all samples examined (n = 19, m.v. 49%, range 15–92%, Table 2). Ten genera of this family were identified. Among them, *Lotus* and *Melilotus* were predominant (>45% in some samples), while *Trifolium repens* type and *Vicia* were secondary (Table 2 and Table S5A). Other important families were the Asteraceae, Boraginaceae, Brassicaceae, and Rosaceae. Notable was the Boraginaceae family, observed in 15 out of 19 honey samples. Five plant genera of this family were identified, and among them, *Echium* and *Cynoglossum/Cerinthe* were classified as secondary pollen (16–45%) in some samples (Table 2 and Table S5A). Other genera with secondary pollen (at least in one sample) were *Sinapis/Brassica* and *Draba/Eruca* (Brassicaceae), *Cirsium* and *Centaurea solstitialis* type (Asteraceae), *Ferula-Scandix* (Apiaceae). All other plant species found in citrus honey samples were in the range of 3–16% (important minor pollen) or <3% (minor pollen) (Table S5A,B).

Among the nectarless plants, 23 families were represented in the Sicily citrus honey samples (Table S5A). The most important families were the following (in brackets the corresponding genera): Oleaceae (*Olea, Fraxinus*), Papaveraceae, Fagaceae (*Quercus coccifera, Q. ithaburensis*), Anacardiaceae (*Pistacia*), Fumariaceae (*Hypecoum, Fumaria*), Cistaceae, and Ranunculaceae (*Adonis, Ranunculus, Anemone*). Of these, the genera *Papaver, Olea, Fraxinus, Quercus,* and *Pistacia* were classified as secondary pollen (16–45% of all pollen types) at least in one sample, while the genera *Hypecoum, Adonis, Cistus,* and *Vitis* (family Vitaceae) were measured as important minor pollen (3–16% of all pollen types) in some of the samples. Pollen grains of plant species from the families Chenopodiaceae/Amaranthaceae, Pinaceae, and Poaceae (Graminae) were encountered in most of the samples (58–74% of the samples), in low frequencies (≤1% of all pollen types, Table S5A,B). The average percentage of nectarless plants was relatively low (m.v. 25%, range 5–40%, Table S5A). The whole pollen spectrum, observed in all honey samples (n = 19), was consistent with the flora of Sicily (for the flora of Sicily, see also Supplementary Materials and [101]). 

**Figure 8:**
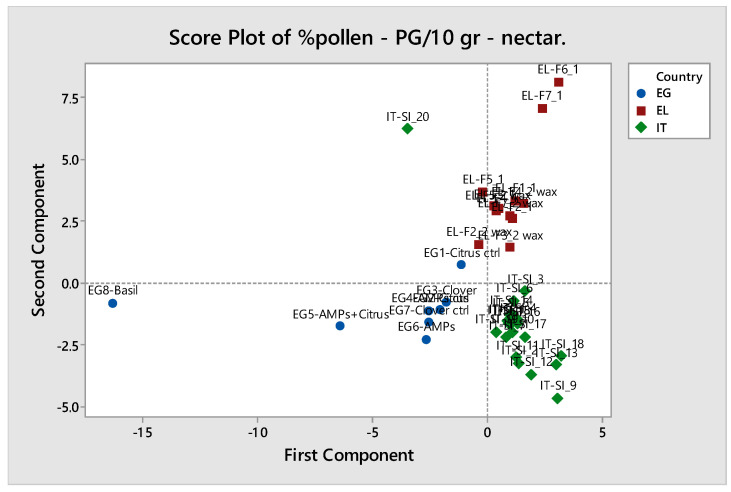
Principal component analysis of honey samples (n = 40) using melissopalynological parameters (listed in detail in Table S5A). Score plots of the samples in the space of the two first principal components.

In the quantitative melissopalynological analysis, 75% of the samples provided results compatible with the class of unifloral honeys with under-represented pollen (*Class I: N* ≤ 20 × 10^3^), while the rest of the samples were measured in *Class II* (*N* = 21 × 10^3^–100 × 10^3^), where *N* is the number of plant elements in 10 g honey (Table S5B). In the specific samples, *N* is in fact the number of pollen grains, as no significant amount of honeydew elements was detected [99]. Finally, the honey samples from the Italian market (n = 12) did not differ significantly from the Italian honeycomb honeys (n = 8), except for one that was excluded from statistical analysis (Figure 8). Nevertheless, a great variety of plant species was observed in citrus honeys from the fields (Table 2 and Table S5A,B).

**Table 2:** Melissopalynological analysis of citrus honey samples from Italy–Sicily. Plant families of which the sum of pollen accounts for 95–100% of the whole pollen spectra of nectariferous plants, expressed as relative pollen frequencies (% of nectariferous plants).

Family	Genus/ Species ^(*)^							HONEY	HONEY	HONEYCOMB HONEY	HONEYCOMB HONEY	HONEYCOMB HONEY	HONEYCOMB HONEY	HONEYCOMB HONEY	HONEYCOMB HONEY									
		Citrus-market	Citrus-market	Citrus-market	Citrus-market	Citrus-market	Citrus-market	Chiaramonte Gulfi (RG)	Chiaramonte Gulfi (RG)	Ispica (RG)	Ispica (RG)	Ispica (RG)	Ispica (RG)	Ispica (RG)	Chiaramonte Gulfi (RG)	Citrus-market	Citrus-market	Citrus-market	Citrus-market	Citrus-market				
		CH_IT SI_1	CH_IT SI_2	CH_IT SI_3	CH_IT SI_4	CH_IT SI_5	CH_IT SI_6	CH_IT SI_7	CH_IT SI_8	CH_IT SI_9	CH_IT SI_10	CH_IT SI_11	CH_IT SI_12	CH_IT SI_13	CH_IT SI_14	CH_IT SI_15	CH_IT SI_16	CH_IT SI_17	CH_IT SI_18	CH_IT SI_19	Count	Mean	Min	Max
Fabaceae	(F1)	67	32	25	41	47	15	69	68	32	35	82	59	36	66	23	52	59	34	92	19	49	15	92
Brassicaceae	(B1)	9	6	24	6	33	3	14	16	5	21	2	9	22	14	18	4	2	12	3	19	12	2	33
Rutaceae	**Citrus**	9	10	31	4	3	18	4	2	3	2	2		<1	5	3	12	1	5	1	18	6	<1	31
Rosaceae	(R1)	1	2	1	1	1	<1	6	4	3	2	2	4	8	2	4		4	1	1	18	3	<1	8
Asteraceae	(A1)	7	9	2	1	4	19	2	4	10	5		15	29	3	8	11	1	6		17	8	1	29
Boraginaceae	(B2)	3	33	12	44	9	25	2		26	5	<1				6	5	3	8	1	15	12	<1	44
Lamiaceae	(L1)		4	2	1					8	3		3	2		2		<1	3		10	3	<1	8
Myrtaceae	Myrtus, Eucalyptus	1		1							3	7	1	1		24		6	19		9	7	1	24
Apiaceae	(A2)		2				5				11			<1		7	6	17	8	2	9	7	<1	17
Ranunculaceae	Clematis					3				3	11	1				1	1		1		7	3	1	11
Fagaceae	Castanea						10						1								2	6	1	10
Lythraceae	Lythrum									8		2									2	5	2	8
SUM		96	98	97	98	100	95	98	94	98	97	98	94	98	90	97	92	93	97	100				

^(*)^ The genus/species of plants referred to in the text and tables correspond to pollen phenotypes encountered in the honey samples and may not be the exclusive representatives of the specific pollen phenotypes. (A1): Cirsium *^2^, Centaurea solstitialis, Anthemis, Taraxacum, Dittrichia/Inula, Centaurea redempta/raphanina, Carthamus. (A2): Ferula-Scandix *^2^, Daucus-Crithmum-Foeniculum, Tordyllium, Smyrnium. (B1): Sinapis/Brassica *^2^, Draba, Eruca *^2^. (B2): Echium *^2^, Cynoglossum creticum *^2^, Cynoglossum/Cerinthe *^2^, Myosotis, Borago. (F1): Lotus corniculatus *^1^, Melilotus officinalis *^1^, Trifolium repens *^2^, Vicia*^2^, Trifolium incarnatum, Ononis spinosa, Robinia, Trifolium pratense, Medicago, Acacia. (L1): Thymbra/Thymus, Ballota, Mentha, Teucrium, Stachys, Rosmarinus. (R1): Rubus, Pyrus/Prunus (genera are presented in descending order of occurrence). *^1^: >45% at least in one sample. *^2^: 16–45% at least in one sample.

#### 2.3.2. Melissopalynological Analysis of Citrus Honey from Argos, Greece

In citrus honey from Argos, Greece (n = 12), the melissopalynological analysis revealed 51 plant families (28 of nectariferous and 23 of nectarless plants, Table S5A). The families of which the sum of pollen accounted for 96–100% of the whole pollen spectra of nectariferous plants, are shown in Table 3. 

Most of the families are common to those encountered in citrus honeys from Italy. However, notable differences exist, such as the relative abundance of each family and the number of plant species per family. The pollen of genus *Citrus* is considered under-represented in honey [12,99]. Nevertheless, in citrus honeys from Argos, it was classified as predominant pollen (m.v. 53%, range 17–79%, Table 3). 

This is mainly due to the absence of other nectariferous plants of the same flowering period as the orange trees, but the variety of orange trees in the field can also interplay. Another fact that affects the relative frequencies is the large percentage of pollen of nectarless plants (m.v. 84%, range 68–91%, Table S5A,B), which is excluded during the calculation of the frequencies of nectariferous plants. Apart from the dominant Rutaceae family (*Citrus*), other important families were the Brassicaceae, Asteraceae, and Boraginaceae families (Table 3). 

The genus *Sinapis/Brassica* (Brassicaceae) was classified as secondary in one-third of the samples, and the genus *Echium* (the only representative of the Boraginaceae family in Greek citrus honey samples) was secondary or even dominant in some samples. Anthemis and Taraxacum pollen types were the most abundant in the Asteraceae family. Other plant species with a high relative frequency (dominant or secondary, at least in one sample) were the Pyrus-Prunus type (Rosaceae) and Tordyllium type (Apiaceae) (Table 3 and Table S5A). 

Among the 23 families of nectarless plants encountered in Greek citrus honeys, the most important were the following (in brackets the corresponding genera): Fagaceae (*Quercus coccifera* type), Oleaceae (*Olea*), Anacardiaceae (*Pistacia*), and Papaveraceae. The genera *Quercus* and *Olea* were dominant (>45% of all pollen types) in several samples. The average percentage of nectarless plants was high (m.v. 84%, range 68–91%, Table S5A,B). The whole pollen spectrum, observed in all honey samples (n = 12), was consistent with the local flora [102,103].

**Table 3:** Melissopalynological analysis of citrus honey samples from Greece–Argos. Plant families of which the sum of pollen accounts for 96–100% of the whole pollen spectra of nectariferous plants, expressed as relative pollen frequencies (% of nectariferous plants).

Family	Genus/Species ^(*)^			Honey-Wax		Honey-Wax		Honey-Wax		Honey-Wax			Honey-Wax				
		FIELD_1_001	FIELD_2_001	FIELD_2_002	FIELD_3_001	FIELD_3_002	FIELD_4_001	FIELD_4_002	FIELD_5_001	FIELD_5_002	FIELD_6_001	FIELD_7_001	FIELD_7_002	Count	Mean	Min	Max
Rutaceae	**Citrus** *^1^	17	66	35	79	63	66	75	36	67	24	33	73	12	53	17	79
Brassicaceae	(B1)	40	20	7	1	1	9	8	8	13	18	24	9	12	13	1	40
Asteraceae	(A1)	5	5	4	13		16	4	6	3	1	13	14	11	8	1	16
Boraginaceae	(B2)	7		4	3	33	2	2	46		24	4		9	14	2	46
Apiaceae	(A2)	<1	7	47	<1	2	<1		<1		1	2		9	7	<1	47
Lamiaceae	(L1)	<1	<1		1	1	1	4			3	7		8	2	<1	7
Fabaceae	(F1)	7	1				1			8	3	2		6	4	1	8
Ericaceae	Erica, Arbutus	2			1					4	3		3	5	3	1	4
Rosaceae	(R1)	17	<1							2		1		4	5	<1	17
Alliaceae	Allium				1				2	<1	6			3	3	1	6
Liliaceae		2	<1		<1		1							4	1	<1	2
Fagaceae	Castanea			2			3					7		3	4	2	7
Myrtaceae	Eucalyptus			2						1	6			3	3	1	6
Ranunculaceae	Clematis		<1						<1		6			3	2	<1	6
Araliaceae	Hedera										6	3		2	5	3	6
Asparagaceae	Asparagus						1	7						2	4	1	7
SUM		96	99	100	99	100	99	100	99	97	99	98	100				

^(*)^ The genus/species of plants referred to in the text and tables correspond to pollen phenotypes encountered in the honey samples and may not be the exclusive representatives of the specific pollen phenotypes. (A1): Anthemis, Taraxacum, Onopordum, Dittrichia/Inula, Cirsium. (A2): Tordyllium *^1^, Ferula-Scandix. (B1): Sinapis/Brassica *^2^, Draba/Eruca. (B2): Echium *^2^, Echium italicum *^1^. (F1): Trifolium repens, Lotus corniculatus, Ononis pubescens, Vicia, Trifolium pratens. (L1): Phlomis/Lamium, Rosemarinus, Teucrium, Thymbra/Thymus. (R1): Pyrus/Prunus *^2^, Rubus (genera are presented in descending order of occurrence). *^1^: >45% at least in one sample. *^2^: 16–45% at least in one sample.

In the quantitative melissopalynological analysis, only one sample was classified as *Class I* (≤20 × 10^3^), nine samples as *Class II* (21 × 10^3^–100 × 10^3^), and one sample was classified as *Class III* (101 × 10^3^–500 × 10^3^) [99]. The under-represented nature of citrus pollen could be confirmed only if the pollen of nectariferous plants was taken into account (see PG-nectariferous/10 g, m.v. 6145, range 2480–9118, Table S5B). This is due to a large number of pollen grains of nectarless plants that do not actually contribute to the formation of honey, but they come from pollen already stored in the honeycomb by the bees. Finally, the relative frequencies of pollen, measured in the seven honey samples and the five honeycomb samples, were quite different, indicating that the presence of honeycomb can affect to a great extent the results of melissopalynological analysis (Table 3 and Table S5A,B). 

#### 2.3.3. Melissopalynological Analysis of Citrus Honey from Al Shaeir Island, Egypt

In honeys from Egypt (n = 8), the melissopalynological analysis revealed the existence of 34 families (19 of nectariferous and 15 of nectarless plants, Table S5A). Although five honeys were declared as unifloral (2 citrus honeys, 2 clover honeys, and 1 basil honey) and three honeys as polyfloral; mainly from aromatic medicinal plants and citrus trees, the most important families were common to all samples (Table 4). 

Despite the under-represented nature of *Citrus* pollen, its relative abundance in the two citrus honeys was high (79% and 29%, respectively, Table 4). In the two clover honeys, the relative frequency of *Trifolium* sp. pollen was 83% and 25% and finally the relative frequency of *Ocinum* pollen in the basil honey was <1% (Table 4). This could be explained by the fact that basil flowers, although of great apicultural value, display a poor pollen collection by bees [104]. 

The most important families, of which the sum of pollen accounted for 95–100% of the whole pollen spectra of nectariferous plants, are shown in Table 4. Apart from Fabaceae and Rutaceae family, expected to be observed in high relative frequencies, as *Trifolium* sp. and *Citrus* trees are cultivated in the specific fields, notable was the Myrtaceae family. In half of the samples, Eucalyptus pollen was dominant (>45%), and in two samples was secondary pollen (16–45%). From the Brassicaceae family, *Lepidium/Draba* type was dominant pollen in one sample and secondary in another. The Malvaceae family, although in low relative frequencies (<2%), was observed in 6 out of 8 samples, represented by more than three plant genera (*Bombax*, *Abutilon*, *Sida*, etc.). The only representative of Lamiaceae family was genus *Ocinum*, encountered in several samples, though in low frequencies (<1%). Finally, the Asparagaceae family was observed in one sample as dominant pollen (Table 4 and Table S5A,B).

**Table 4:** Melissopalynological analysis of citrus honey samples from Egypt–Al Shaeir Island. Plant families of which the sum of pollen accounts for 95–100% of the whole pollen spectra of nectariferous plants, expressed as relative pollen frequencies (% of nectariferous plants).

Family	Genus/Species ^(*)^	HBc-Control Citrus	HB1-CF Citrus	HB2-CF	HBc-AMPs Control	HB1-AMP + Citrus	HB2-AMP	HB1-Clover Control	HB1-Basil				
		EG1-Citrus	EG2-Citrus	EG3-Clover	EG4-AMPs	EG5-Basil + Citrus	EG6-Basil + Borago+ Coriandrum+ Anethum + Carum	EG7-Clover	EG8-Basil	Count	Mean	Min	Max
Fabaceae	(F1)	3	1	25	2	<1	66	83	2	8	23	<1	83
Myrtaceae	Eucalyptus *^1^, Myrtus/Psidium	3	62	50		57	26	16	46	7	37	3	62
Rutaceae	**Citrus** *^1^	79	29	18	1	6	<1	<1		7	19	<1	79
Brassicaceae	(B1)	3	4	1	91	24	3			6	21	1	91
Apiaceae	(A2)	2	2	7		6	1	<1		6	3	<1	7
Malvaceae	(M1)	<1	<1	<1		2	<1		<1	6	<1	<1	2
Lamiaceae	Ocinum	<1			<1	<1	<1		<1	5	<1	<1	<1
Asteraceae	(A1)	7					1		5	3	4	1	7
Portulacaceae	Portulaca				5			<1	<1	3	5	5	5
Asparagaceae	Asparagus/Other *^1^								47	1	47	47	47
SUM		98	99	100	99	95	96	99	98				

^(*)^ The genus/species of plants referred to in the text and tables correspond to pollen phenotypes encountered in the honey samples. They are consistent with the crops of the field and the local flora and may not be the exclusive representatives of the specific pollen phenotypes. (A1): Helianthus, Dittrichia/Inula, Taraxacum, Anthemis. (A2): Coriandrum, Foeniculum, and other plants of the Apiaceae family. (B1): Lepidium/Draba/Eruca *^1^, Sinapis/Brassica. (F1): Trifolium pratense *^1^, Trifolium repens, Vicia, Delonix regia. (M1): Bombax, Abutilon, Sida, Other (genera are presented in descending order of occurrence). *^1^: >45% at least in one sample. *^2^: 16–45% at least in one sample.

Among the 17 families of nectarless plants, the most important were the following: Poaceae (Graminae), Arecaceae (Palmae), Solanaceae, Cyperaceae, Asteraceae, Chenopodiaceae/Amaranthaceae, Cistaceae, and Oleaceae. The Arecaceae (Palmae) family was observed as secondary or important minor pollen in several samples and the Asteraceae family (*Artemisia* and *Xanthium* genus) was dominant pollen in the basil honey. The average percentage of nectarless plants was relatively low (m.v. 23%, range 1–64% of all pollen types, Table S5A,B). The whole pollen spectrum, observed in all honey samples (n = 8), was consistent with the local flora and the plants cultivated in the specific fields [105].

Notable differences from the Italian and Greek honeys were the strong presence of the Myrtaceae family, the various genera of the Malvaceae family and the predominance of nectarless plant families different from those of the Italian and Greek samples, especially the Arecaceae (Palmae) family (Table S5A). 

Quantitative melissopalynological analysis provided contradictory results, as a large variation was observed: one sample was classified in *Class I* (*N* ≤ 20 × 10^3^), two samples in *Class II* (*N* = 21 × 10^3^–100 × 10^3^), three samples in *Class III* (*N* = 101 × 10^3^–500 × 10^3^) and two samples in *Class V* (*N* > 10^6^). A possible explanation for this, is that honey was extracted from the honeycomb by pressing or squeezing, thus making possible the enrichment of honey with pollen stored in the honeycomb by the bees, possibly in cells blocked by a quantity of honey. 

**Figure 9:**
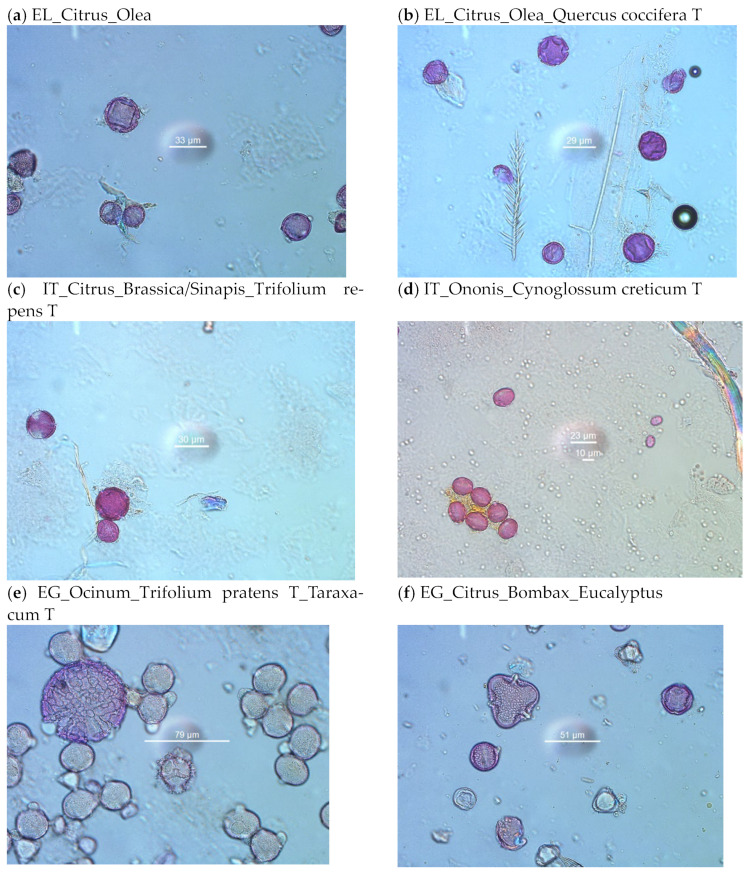
Indicative pollen grains in Greek (EL, **a**,**b**), Italian (IT, **c**,**d**), and Egyptian (EG, **e**,**f**) honey.

Overall, citrus honeys from the three different countries (Italy, Greece, and Egypt), as well as honeys of different botanical origins from Egypt, showed a different melissopalynological profile, which made it possible to distinguish the samples using principal component analysis (Figure 8 and Figure 9). With the exception of one sample from Italy (CH-IT-20) and the “basil honey” from Egypt, all other samples were well grouped.

### 2.4. Environmental Factors

The biosynthesis and accumulation of plant secondary metabolites (PSMs) are governed by inherent and extrinsic factors. The latter encompass biotic and abiotic environmental drivers such as the response to parasites, insects, light irradiation, temperature, water content on soil, etc. [106]. 

Research has shown that the selectivity of biosynthetic reaction pathways in plants is influenced by seasonal variations reporting differences in flavonoid levels and content [107]. Despite not the scope of the present research, environmental parameters were contemplated in this work in an effort to explain the obtained results. 

Annual mean temperatures, humidity, rainfall, sunlight, etc., were acquired from the nearest meteorological station or from the National Meteorological Data Service Centre of Greece, Italy, and Egypt. The latter provides the most precise data for the respective regions. A long period of light irradiation was reported to increase the concentrations of PSMs, such as flavonoids and phenolic acids. Light intensity is also a parameter not to disregard. 

Nevertheless, the three Mediterranean regions share common weather features, adding complexity to the challenging task of attributing specific chemicals’ relative increase or decrease to environmental factors. For the Argolida region in Greece, an average temperature of 15.4 °C and relative humidity of 67.1% was recorded for March–May 2020 (total annual sunshine reaching 2561 h per year was also documented). The solar radiation observed in the area (1700–1800 kWh/m^2^/y) is higher in Greece. In Sicily (Csa, hot summer Mediterranean climate), where 8 experimental plots were established, the annual sunshine reaches 2700 h that is 1.05 times higher than the respective in Argolida Greece. The respective regions in Egypt are similarly characterized by a hot desert climate (BWh) which is extremely dry with virtually no rainfall. Annual sunshine in Egypt is higher than in Greece and Italy, with 3300 h in the upper part (close to the Mediterranean). More specifically, in Egyptian pilot areas the period March to May 2020 an average temperature of 21.7 °C, and a relative humidity of 49.3% were recorded. Based on the literature long photoperiod increases the biosynthesis and concentrations of flavonoids and phenolic acids, compared to short durations of light irradiation [106]. Flavonoids and phenolic acids were less expressed than in Italy and Greece. However, for Egypt, the latter was not verified, a fact than can also be attributed to the lower number of Egyptian citrus honey integrated into the specific study, and the less diverse flora of the Egyptian pilot areas. On the other hand, the metabolomics approach to Greek and Italian honey might postulate that apart from the citrus tree varieties, the higher average sunshine in Sicily might have favored the formation of more flavonoids and phenolic acids than in Greece, considering that both areas were rich in AMPs. On the contrary, terpenic molecules are constantly differentially increased in Greek honey. The latter can possibly be attributed to the more shaded conditions and lesser sunshine that preponderate in the region compared to Italian region, which is reported to favor the formation of some terpenes [108]. Nevertheless, such a conclusion needs careful consideration since it cannot be regarded as a generic rule for all terpenes since studies showed that not all terpenes biosynthesis are affected the same way under comparable environmental conditions [109]. In addition, it reflects the specific honey samples collected during 2020, whose chemical portfolio through the biosynthesis of all classes of plant secondary metabolites (terpenes, phenolic and nitrogen-containing compounds) is shaped predominantly by the citrus crop, the flora of the region and the climatic conditions. 

Since the case study areas are not homologous, though most of them refer to the predominating citrus crop and derived citrus honey, it is risky to further elaborate on how other parameters (solar radiation in connection to altitude) affected the generation of PSMs. Nevertheless, this work adds another piece of evidence on the multifactorial issue of PSMs biosynthesis and the variations in the diversity of compounds and their concentrations.

A deeper analysis of individual flavonoids, phenolic acids, terpenoids, and other chemicals’ concentrations and variations will continue to further reinforce the presented findings. To build upon this work, additional honey samples from the two succeeding years (2021 and 2022, beehives were also placed in the same case study areas) of the PLANT-B project will be incorporated, and the impact of installed AMPs might be more evident-traceable in honey’s composition. The latter will be viewed under an overall metabolomics scheme along with prospective and solid quantification of not yet identified or reported bioactive compounds. 

## 3. Materials and Methods

### 3.1. Chemicals and Reagents

The analytical standards used in the specific study (>90% purity) were the following: Caffeic acid, caffeic acid phenethyl ester (CAPE), chrysin, luteolin, daidzein, suberic acid, apigenin, (Alfa Aesar, Kandel, Germany), pinocembrin, isorhamnetin, isosakuranetin, vitexin, orientin, rosmarinic acid, myricetin, vanillin, ursolic acid, hydroxytyrosol, tangeretin, chrysoeriol, betulinic acid, eriodictyol, sakuranetin, naringenin, *t*-cinnamic acid, genistein, diosmetin, resveratrol, galangin, pinocembrin 7-methyl ether, techtochrysin, (Extrasynthese, Genay, France) rutin, isoferulic acid, ellagic acid, kaempferol, quercetin, corosolic acid, acacetin, diosmin, protocatechuic acid ethyl ester, hesperetin, phloridzin, chlorogenic acid, *p*-coumaric acid, (±)catechin, narirutin, (+)-abscisic acid (Sigma Aldrich, Seelze, Germany), rhamnetin, syringic acid, protocatechuic acid, ferulic acid, kaempferide, adipic acid, pinostrobin, gallic acid, pinobanksin, caffeine, (Fluka, Seelze, Germany), naringin, hesperidin, and scopoletin (Acros Organics, Geel, Belgium) pinobanksin-3*O*-acetate (Interchim Inc., Los Angeles, CA, USA), cinnamylidenacetic acid (Wako Chemicals, Osaka, Japan), *O*-οrselllinaldehyde (Santa Cruz Biotechnology, Dallas, TX, USA) and hispidulin, quercetin-3-O-sophoroside from Cayman Chemicals (Ann Arbor, MI, USA). Synephrine was obtained from TCI Chemicals (Tokyo, Japan). 

Acetonitrile (ACN), methanol (MeOH), and formic acid of liquid chromatography-mass spectrometry (LCMS) grade were obtained from Merck (Darmstadt, Germany). Ultrapure water (H_2_O) was produced from the SG Millipore apparatus. Discovery® octadecylsilane (DSC-C_18_) solid phase extraction (SPE) cartridges (500 mg, 6 mL) were purchased from Sigma Aldrich (Supelco, Bellefonte, PA, USA). Nylon filters (0.22 μm) were obtained from Macherey-Nagel (Duren, Germany). All reagents and chemicals were of analytical grade. 

### 3.2. Honey Samples

A total of 28 samples were collected from all countries: Greece (7 honey + 5 replicate honeycomb honey), Italy (8 honeycomb honey), and Egypt (8 honey). To reinforce the metabolomics approach, 12 citrus honey samples from the Italian market were purchased and included in the study. The majority of samples were citrus honey, yet some honey samples were produced exclusively from AMPs orchards. More specifically, in Egypt, two fields featured citrus in addition to clover in one of them, and the second field had clover in addition to the combination of basil, borage, coriander, anise, and caraway. In Greece and Italy, all samples were derived from citrus orchards, some of them containing installed AMPs.

In Greece, 7 experimental orange orchards, *cv* Navelina and Merlin, were established in the region of Argolida (see Figure S1). In each field, five 10-frame Langstroth hives beehives were placed. The beehives were placed on pallets. The bee colony hives were of the same honeybee population, with queens of the same age, and had been handled in the same way during the winter and spring. The installation of the beehives in the experimental fields occurred on 23 April 2020, while the orange trees were at the beginning of flowering (5–10%) in most orchards and in 30–50% of full flowering in orchards of early blooming. The beehives were inspected every week, and the necessary beekeeping manipulations were carried out (addition of frames, swarming control, expanding space for honey and egg laying, etc.) and were recorded. The honey harvest was made on 14 May, 3 weeks later and while the orange trees were in 50–100% of full flowering.

The frames with the ripe honey placed on empty floors were transferred on the same day to the Laboratory of Apiculture of the Institute of Mediterranean Forest Ecosystems and Forest Products Technology (Greece), and the next day the extraction of honey occurred. Two kinds of samples were collected, honey samples from the honey extract and honeycomb samples with honey. The pieces of honeycomb with sealed honey were placed in glass jars. Honey from the honeycombs was obtained at Benaki Phytopathological Institute (Greece) by squeezing, and the honey was strained by gravity into a clean container. 

In the frames that were placed in the honey extractor, wax cappings were removed with a cold scratcher, and the honey was also strained by gravity from a stainless strainer to a honey tank. The honey was then allowed to rest for at least one week, during which time all air bubbles present in the honey floated to the top. Then pure honey was drained through a latch at the bottom of the tank to fill glass jars. All samples were placed in a freezer at −18 °C until the day of their analysis.

In Italy, the Sicilian experimental orange orchards (Figure S1) contained the Navel orange variety, with the exception of the experimental sites in the municipality of Chiaramonte Gulfi (IPM3, O1, and O2) that contained the “Tarocco” orange varieties. Honey sampled during the first year of experimentation was derived from Italian-Dadant hives with 10 and 6 frames. The supers were removed by preparing the bee escapees 24 h before. Subsequently, the wax capping was removed, and the frames were centrifuged. The extracted honey was filtered and decanted. Occasionally, some hives had not filled the supers, and therefore, the honey was recovered directly from a portion of the honeycomb. Egyptian experimental orange orchards (Figure S1) also contained Navel oranges. The complete information on samples, location, and installed AMPs are presented in Figure S1 and Table S1 (Supplementary Material).

### 3.3. Sample Preparation

Honey (3 g) was homogenized with the aid of a glass rod in 7 mL of acidified ultrapure H_2_O (pH = 2.2). Then, the mixture was loaded on a pre-activated DSC-C18 SPE cartridge (activation was performed by succeeding elution of MeOH (3 mL) and H_2_O (3 mL)). Consequently, the SPE cartridge was washed with acidified H_2_O (2 mL) and 5 mL of ultrapure H_2_O. Compounds of interest were eluted with 3.5 mL of a MeOH:ACN (2:1, *v*/*v*) solution. Then, the eluate was evaporated to dryness using a rotary evaporator (bath temperature not surpassing 30 °C) and reconstituted in MeOH:H_2_O (7:3, *v*/*v*). After filtration with nylon filters (0.22 μm), the extract was injected into LC-HRMS. A pooled quality control (QC) sample was prepared by transferring 10 μL of each sample to an LC vial to assess system stability throughout the batch analysis.

### 3.4. Ultra-High-Performance Liquid Chromatography Coupled to Orbitrap High-Resolution Mass Spectrometry Analysis of Honey Extracts

UHPLC was performed using a Dionex Ultimate 3000 UHPLC system (Thermo Scientific, Karlsruhe, Germany). A Hypersil Gold UPLC C18 (2.1 × 150 mm, 1.9 μm) reversed phased column (Thermo Scientific, Germany) was used for the separation of the analytes. The analysis was performed on a Q-Exactive Orbitrap mass spectrometer using a negative and positive heating electrospray ionization source (Thermo Scientific, Germany).

The mobile phase consisted of solvents A: aqueous 0.1% (*v*/*v*) formic acid and B: MeOH. A gradient elution methodology from 0 to 40 min has been employed as follows: T = 0 min, 20%B; T = 2 min, 20%B; T = 12 min, 70%B, T = 32 min, 95%B, T = 37 min, 95%B, T = 37.1 min, 20%B; T = 40 min, 20%B. The flow rate was 0.220 mL/min, and the injection volume was 5 μL. The column temperature was kept at 35 °C while the sample tray temperature was set at 10 °C. 

The optimized conditions for analysis were set as follows: capillary temperature, 350 °C; spray voltage, 2.7 kV (for negative) and 4 kV (for positive); S-lense Rf level, 50 V; sheath gas flow, 40 arb. units; aux gas flow, 5 arb. units; aux. gas heater temperature, 50 °C. Analysis was performed using the Fourier transform mass spectrometry mode of the LTQ orbitrap (FTMS) in the full scan ion mode, applying a resolution of 70,000, while the acquisition of the mass spectra was performed in every case using the centroid mode. The mass range for full MS was set at 120–1200 *m*/*z*. The data-dependent acquisition capability has been used at 35,000 resolution, allowing for MS/MS fragmentation of the three most intense ions of every peak applying a 10 s dynamic exclusion. Stepped normalized collision energy was set at 40, 60, and 100. The Xcalibur version 4 was used for data acquisition. A pooled QC sample was analyzed three times in the beginning and three times at the end of the acquisition, and every after six samples.

### 3.5. High-Performance Liquid Chromatography Photo Diode Array Electrospray Mass Spectrometry (HPLC-PDA-ESI/MS)

A Shimadzu (Kyoto, Japan) LCMS-2010 EV Liquid Chromatograph Mass Spectrometer instrument was used with the LCMS solution version 3.0 software consisting of a SIL-20A prominence autosampler and an SPD-M20A diode array detector. The latter were coupled in series with a mass selective detector equipped with an atmospheric pressure ionization. The LC separation was achieved on a Zorbax Eclipse Plus, 3.5 μm, 150 × 2.6 mm i.d. chromatographic column (Agilent Technologies, Santa Clara, CA, USA). The mobile phase consisted of two channels, channel: 0.1% formic acid in water (A) and ACN (B). The flow rate and mobile phase gradient were identical to previously published work [110]. Similarly, the validation of the analytical method and quantitation of analytes was grounded on the previous work of our group [110], adapting the same sample preparation followed for the UHPLC-HRMS analysis. 

### 3.6. GC-MS Analysis

#### SPME Holder and Fibers

The SPME holder and coated fibers (85 μm PolyAcrylate (PA), 100 μm Polydimethylsiloxane (PDMS), and 65 μm CarboWax/divinylbenzene (CW/DVB)) were supplied by Supelco (Bellefonte, PA, USA). For honey extraction, PA fiber was selected. More specifically, 1 g of honey was mixed with 5 mL 10% NaCl, shaken in a vortex mixer for 1 min, placed into a 9 mL headspace vial (Alltech, Alltech Ass. Deerfield, IL, USA), and positioned in an aluminum block heater. After 30 min of the preheating period at 60 °C with simultaneous stirring, the SPME needle penetrated the vial septum, and the fiber was exposed in the headspace of the solution. Sampling was performed for an additional 20 min at 100 °C, and finally, the needle was removed from the vial and injected into the heated injection port of the gas chromatograph. A Shimadzu Nexis GC 2030 gas chromatograph (Shimadzu Corporation, Kyoto, Japan) equipped with an AOC-6000 autosampler and a Shimadzu GCMS-TQ8040 NX triple quadrupole. Data acquisition and processing were performed by LabSolutions GCMS solution software, version 4.52. Desorption was allowed for 5 min. The injector port temperature was kept at 250 °C. A MEGA 5-HT (MEGA S.r.l., Legnano, Italy) column (30 m length × 0.25 mm i.d. × 0.25 µm film thickness) was used. The instrument worked at a constant flow of 1.55 mL/min, using the full scan mode (range of 50–500 amu) and helium (99.999% purity) as the carrier gas. The GC oven program was the following: Initial temperature at 40 °C (stayed for 3 min), ramped linearly to 260 °C, at a rate of 5 °C/min, where it stayed for an additional 8 min (overall runtime 55 min). 

### 3.7. Data Post-Processing and Chemometrics Analysis

Raw data were exported to Compound Discoverer 2.1 (Thermo Fisher Scientific Inc., San Jose, CA, USA) for peak detection, deconvolution, deisotoping, alignment, gap filling, and composition prediction procedures. Peaks in which the quality control (QC) sample coverage was less than 50% and the relative standard deviation of the areas under the peaks was more than 30% were excluded. The normalization was QC-based, applying the median absolute deviation (MAD) normalization type. Adjusted *p*-values were calculated using the Benjamini–Hochberg to display the statistically significant variables. Log2 fold change values were calculated to show the variation of the selected differential metabolites between groups. The generated peak lists (for positive and negative mode) from Compound Discoverer containing the accurate masses and retention time paired with corresponding intensities for all detected peaks of all the samples were exported as a .csv file, imported to Microsoft Excel 2010 and manipulated appropriately using the “concatenate”, “round” and “transpose” commands. These lists were imported in R to generate heatmaps based on the geographic origin of the honey extracts, using the function heatmap.2 from the Bioconductor package, gplots.

The data were then subjected to multivariate statistical analysis (MVA), i.e., principal component analysis (PCA) and orthogonal projections to latent structures discriminant analysis (OPLS-DA) using the ropls R Bioconductor package [111] in order to determine the optimal number of components (Supplementary Table S6), confirm the validity of the model by permutation testing utilizing 200 random permutations, detect outliers and perform feature selection with variable importance in projection (VIP) scoring from OPLS-DA models. For both PCA and OPLS-DA, different scaling methods were assessed, such as mean-centering only, mean-centering with pareto scaling, and mean-centering with unit variance scaling after log10 data transformation. For both analyses, the standard scaling method was selected as it afforded better clustering between the groups. For the selection of the markers that are differentially increased among the countries, the features that exhibited a VIP scoring greater than 1.5 were further verified by adjusted *p*-value (≤0.05) and Log2 fold change (>1.5). These variables were characterized as the variables that contribute the most to the clustering formation observed in OPLS-DA analysis. 

For the putative annotation of the compounds the mzCloud (https://www.mzcloud.org, accessed on 10 June 2021) database was used applying *m*/*z* tolerance of 5 ppm taking into consideration the isotope distribution similarity and MS/MS fragmentation pattern. 

For the compounds for which there was no record, the online in silico fragmentation tool MetFrag [112] was used, applying 5 ppm search tolerance and 0.001 mass deviation to match generated fragments against MS/MS peaks. For the MetFrag workflow, the candidate structures were retrieved from the databases Kegg (http://www.genome.jp/kegg/compound/, accessed on 10 June 2021), LipidMaps (www.lipidmaps.org, accessed on 10 June 2021), and the Human Metabolome Database (https://hmdb.ca, accessed on 10 June 2021). For the selection of the compounds, a > 0.8 final score was applied.

### 3.8. Melissopalynological Analysis

For the melissopalynological analysis, the methods of Louveaux and von der Ohe were applied [99,113]. In this respect, a solution of 10 g of honey in 20 mL of distilled water was centrifuged for 10 min at 1000× *g*. After decanting the supernatant liquid, the sediment was suspended again in 20 mL of distilled water and centrifuged for 5 min at 1000× *g*. The supernatant liquid was decanted, and the sediment was spread over a microscope slide on a heating plate (40 °C). The sediment is left to dry and then is colored with some drops of a solution of fuchsine (~0.05‰ *w*/*v* in ethanol/water 1/1). After drying (40 °C), a coverslip with a drop of glycerin jelly (mounting medium; Kaiser’s Glycerol Gelatin TM Merck 1.09242.0100) was placed upon the sediment, and the slide was left on the heating plate for another 5 min. The microscope slide is investigated under the microscope (400×). For the determination of relative frequencies of pollen types, 500 to 1000 pollen grains were counted [114]. To detect all possible plant sources, especially those that exhibit very large pollen grains, which are not expected to be present in a substantial amount, the entire slide was scanned at 50× magnification. Quantitative melissopalynological analysis was achieved using Slide Grids, 20 mm × 20 mm. At least 10 mm^2^ were counted. 

The assignment of pollen phenotypes to plant genera or species was accomplished using electronic atlases of pollen (pollen databases) [104,115,116,117,118] and related articles [105,119,120]. It was also confirmed by the project participants through their local collaborators (in Italy, Egypt, and Greece) as well as by plant databases [103,121,122,123]. Especially for the case of Greek honeys, in-house databases were also used. In any case, the genus or species referred to in the text and tables correspond to a pollen phenotype. The multivariate analysis of the melissopalynological analysis results was conducted using Minitab^®^ 17.1.0. Software. A total of 136 variables were used for the 40 samples examined. A number of 39 of the new variables were able to describe 100.0% of the system. 

## 4. Conclusions

The application of an LC-HRMS metabolomics workflow combined with an elaborate melissopalynological analysis managed to unveil several new potential markers of Mediterranean citrus honey potentially associated with citrus crops and the local indigenous flora. Even though most samples were of citrus origin, 40 differentially increased compounds emerged, suggesting a potential effect of both the geographical and botanical origin of the samples. Italian samples were principally characterized by relatively increased levels of flavonoids (such as hispidulin, dihydrokaempferol) and glycerophospholipids, while in Greek samples, selected terpenoids (such as provincialin), iridoid glycosides (e.g., patrinoside), and fatty acids predominated. Egyptian samples showed augmented levels of suberic acid and fatty acyl glycosides. The different varieties of citrus crops, especially between Italy and Greece, seem to shape these differences, subsidized by the local flora and the geographical origin. Concurrently, quantification of common compounds by targeted HPLC-PDA-ESI/MS was also conducted, showing comparable results with the literature. Even though not at the forefront of this work, GC-MS managed to disclose two furan derivatives, never reported, to our knowledge, in citrus honey, as distinguishing chemicals of Greek citrus honey. Consequently, the present work managed to identify molecules that can function as chemical markers of citrus honey, covering a broad area of the Mediterranean basin. Newly reported markers not only better characterize honey but can also help beekeepers to improve the position of their product in a global antagonistic market where quality demands additional scientific evidence. 

## Data Availability

All data available are presented in this manuscript.

## Supplementary Materials

The following supporting information can be downloaded at: https://www.mdpi.com/article/10.3390/molecules28093967/s1, Figure S1: Sampling locations of the PLANT-B case studies in Greece (Argolis, Peloponnese) Egypt (Al Shaeir Island), Italy (Sicily) (Images obtained from Google Earth); Figure S2: Loading plots of Melissopalynological parameters in the space of the two first principal components Table S1A: Egyptian case study honey samples; Table S1B: Italian case study honey samples; Table S1C: Greek case study honey samples and exact location; Table S2: Flavonoids, phenolic and organic acids quantified by HPLC-PDA-ESI/MS in orange blossom honey from the three countries; Table S3: The dominating flora during the citrus flowering season of experimental fields at Al-Qanater Al-Khairiya station–Alshaeir Island–Egypt [124,125]; Table S4: The flora of experimental fields in Sicily, Italy; Table S5A: Qualitative melissopalynological analysis for Greece (EL), Italy (IT), and Egypt (EG); Table S5B: Quantitative melissopalynological analysis for Greece (EL), Italy (IT), and Egypt (EG); Table S6: Parameters from OPLS-DA analysis for each comparison, using the ropls R Bioconductor package. For the analysis, the standard scaling method was used after log10 data transformation.

## Sample Availability

No samples availability.
