# Supplementary material for: Fingerprinting Chemical Markers in the Mediterranean Orange Blossom Honey: UHPLC-HRMS Metabolomics Study Integrating Melissopalynological Analysis, GC-MS and HPLC-PDA-ESI/MS
Source: Molecules. 2023 May 8;28(9):3967. doi: 10.3390/molecules28093967 (PMC10180536; doi:10.3390/molecules28093967)
Supplement: Supplementary file 1 [file molecules-28-03967-s001.zip › molecules-2351556-supplementary.pdf]

Fingerprinting chemical markers in the Mediterranean Orange Blossom Honey: UHPLC-HRMS metabolomics study integrating melissopalynological analysis, GC-MS and HPLC-PDA-ESI/MS

Figure S1: Sampling locations of the PLANT-B case studies in Greece (Argolis, Peloponnese) Egypt (Al Shaeir Island), Italy (Sicily) (Images obtained from Google Earth)

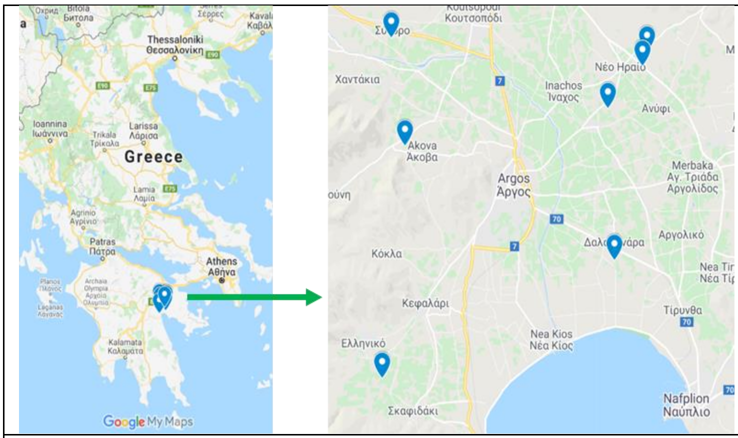

Greece

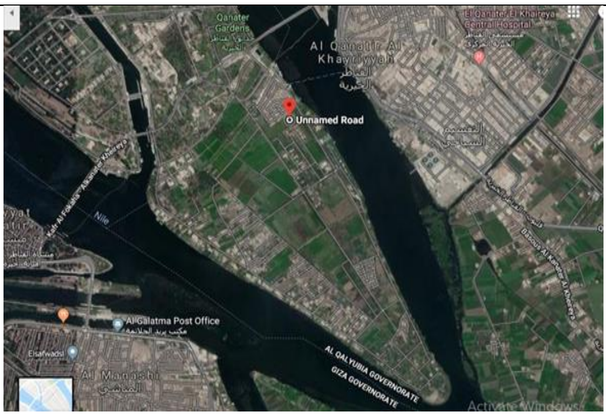

Egypt

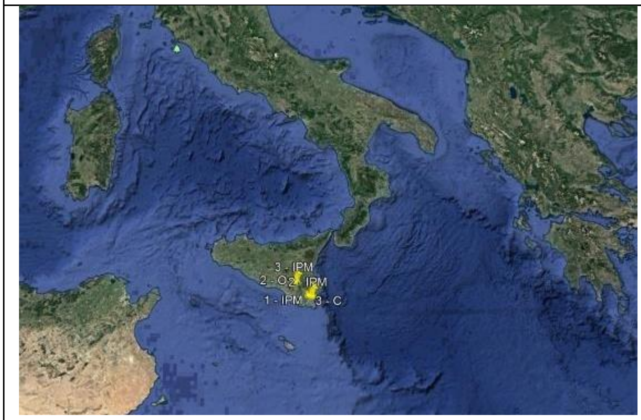

Italy (Sicily)

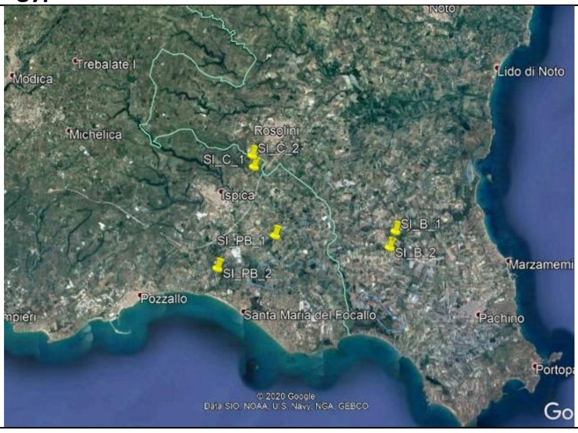

Italy (Sicily)

Table S1A: Egyptian case study honey samples

Samples	Crop	Date (m/y)	Place
Honey	Basil+ Citrus	5/ 2020	Al Shaeir Island
Honey	Citrus	5/ 2020	Al Shaeir Island

Honey	Clover	6/ 2020	Al Shaeir Island
Honey (Control)	Clover	6/ 2020	Al Shaeir Island
Honey (Citrus – Control)	Citrus	5/ 2020	Al Shaeir Island
Honey	Basil	2/ 2020	Al Shaeir Island
Honey	Basil+ *Borag+Coeiandrum+Anethum+Carum*	6/ 2020	Al Shaeir Island
Honey (AMP - Control)	AMPs	6/ 2020	Al Shaeir Island

Table S1B: Italian case study honey samples

HONEY	CITRUS	Chiaramonte Gulfi (RG)	SICILY
HONEY	CITRUS	Chiaramonte Gulfi (RG)	SICILY
HONEYCOMB HONEY	CITRUS	Ispica (RG)	SICILY
HONEYCOMB HONEY	CITRUS	Ispica (RG)	SICILY
HONEYCOMB HONEY	CITRUS	Ispica (RG)	SICILY
HONEYCOMB HONEY	CITRUS	Ispica (RG)	SICILY
HONEYCOMB HONEY	CITRUS	Ispica (RG)	SICILY
HONEYCOMB HONEY	CITRUS	Chiaramonte Gulfi (RG)	SICILY

Table S1C: Greek case study honey samples and exact location

PLANT-B Case study fields (all citrus)	Lat	Long	Sample Number
PLANT-B - Rosemary	37,671857	22,782551	1
BIO Rosemary	37,676275	22,784508	2
PLANT-B - Savory	37,658812	22,768095	3
PLANT-B - Oregano	37,612032	22,770833	4
PLANT-B - Sage	37,57559	22,6732	5
CONTROL - 2	37,647203	22,682517	6
CONTROL - 1	37,680646	22,676654	7

Table S2. Flavonoids, phenolic and organic acids quantified by HPLC-PDA-ESI/MS in orange blossom honey from the three countries

	Mean content µg/g		
**Analyte**	**Italy**[a]	**Greece**[b]	**Egypt**[c]
Apigenin	0.12±0.03	0.10±0.02	0.07±0.01
Quercetin	0.39±0.09	0.34±0.10	0.24±0.07
Galangin	1.21±0.27	1.41±0.15	1.14±0.12
Luteolin	0.21±0.07	0.19±0.05	0.23±0.08

Pinobanksin	2.90±0.25	3.71±0.43	3.05±0.27
Isosakuranetin	2.81±0.24	2.99±0.38	3.09±0.52
Techtochrysin	0.16±0.07	0.18±0.07	0.14±0.05
Protocatechuic acid	10.81±1.09	12.82±2.42	12.24±1.78
Gallic acid	0.08±0.03	0.09±0.02	0.10±0.02
Ferullic acid	1.22±0.28	0.96±0.21	0.65±0.13
trans-cinnamic acid	0.15±0.03	0.22±0.06	0.14±0.04
Syringic acid	12.23±1.31	10.78±0.68	10.01±2.17
p-Coumaric acid	5.38±0.45	6.71±0.71	4.78±0.43
Hesperetin	0.49±0.20	0.19±0.04	0.14±0.04
Hesperidin	1.12±0.16	3.78±0.51	0.71±0.17
Sakuranetin	0.09±0.03	0.05±0.02	0.04±0.01
Chrysin	1.24±0.18	0.98±0.11	0.88±0.09
Naringenin	4.26±0.14	2.96±0.24	0.97±0.06
Pinocembrin	3.38±0.41	2.63±0.27	2.09±0.31
Rutin	5.81±0.35	4.21±0.51	3.72±0.41

a: n=8 , b: n=7, c: n=4

Figure S2. Loading plots of Melissopalynological parameters in the space of the two first principal components

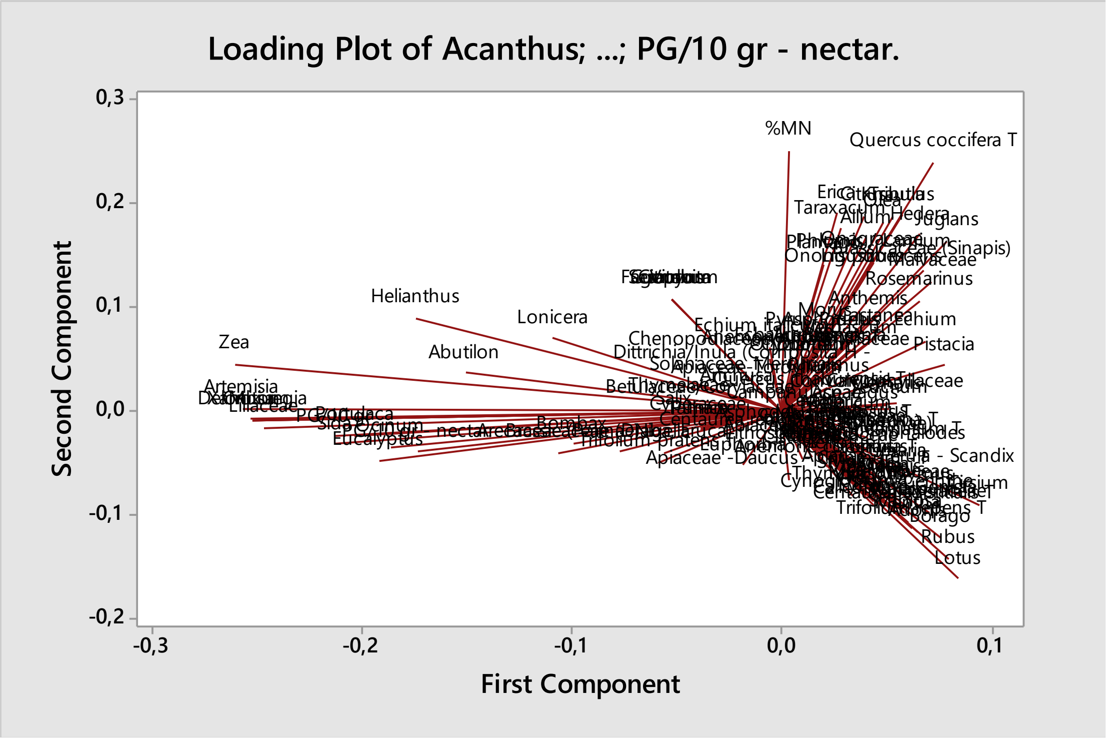

Table S3. The dominating flora during the citrus flowering season of experimental fields at Al-Qanater Al-Khairiya station – Alshaeir Island- Egypt [1,2]

No.	Family	Genus	Species

1	***Rosaceae***	*Prunus*	*Prunus persica* L.
2	***Rosaceae***	*Prunus*	*Prunus armeniaca* L.
3	***Rosaceae***	*Prunus*	*Prunus domestica* L.
4	***Leguminosae***	*Vicia*	*Vicia faba* L.
5	***Leguminosae***	*Pisum*	*Pisum sativum L.*
6	***Boraginaceae***	*Borago*	*Borago officinalis* L.
7	***Lauraceae***	*Cinnamomum*	*Cinnamomum camphora* L.
8	***Oleaceae***	*Jasminum*	*Jasminum grandiflorum* L.
9	***Lamiaceae***	*Ocimum*	*Ocimum basilicum* L.
10	***Compositae***	*Matricaria*	*Matricaria chamomilla* L.
11	***Arecaceae***	= *Phoenix*	*Palma dactylifera* L.
12	***Rosaceae***	*Fragaria*	*Fragaria × ananassa* Duch
13	***Anacardiaceae***	*Mangifera* (	*Mangifera indica* L.
14	***Rosaceae***	*Malus*	*Malus domestica* Borkh.
15	***Myrtaceae***	*Psidium*	*Psidium guajava* L.
16	**Umbelliferae/Apiaceae**	*Petroselinum*	*Petroselinum crispum* Mill.
17	**Umbelliferae/Apiaceae**	Coriandrum	*Coeiandrum sativum* L.
18	***Lythraceae***	*Punica*	*Punica granatum* L.
19	***Myrtaceae***	*Eucalyptus*	*Eucalyptus camaldulensis Dehn.*
20	**Umbelliferae/Apiaceae**	*Anethum*	*Anethum graveolens* L.
21	***Verbenaceae***	*Lantana* (family).	*Lantana camara* L.
22	**Compositae**	Lactuca	*Lactus sativa* L.
23	**Umbelliferae/Apiaceae**	*Ammi* (family *Apiaceae*).	*Ammi visnaga* L.
24	***Solanaceae***	*Solanum* (family).	*Solanum melongena* L.
25	***Solanaceae***	*Capsicum*	*Capsicum sp.* L.
26	**Lamiaceae**	Mentha	*Menthe sp.* L.
27	***Cyperaceae***	*Cyperus*	*Cyperus rotundus* L.
28	***Amaryllidaceae***	*Allium*	*Allium cepa* L.
29	***Cyperaceae***	*Cyperus*	*Cyperus difformis* L.
30	***Convolvulaceae***	*Convolvulus*	*Convolvulus arvensis* L.
31	***Malvaceae***	*Corchorus*	*Corchorus olitorius* L.
32	***Amaranthaceae***	*Beta*	*Beta vulgaris* L.

Table S4. The flora of experimental fields in Sicily, Italy

Family	Genus	Species
**Pinaceae**		
	Pinus	*p. halepensis*
	Pinus	*pinca l.*
**Cupressaceae**		
	Cupressus	*sempervirens*
**Ephedraceae**		
	Ephedra	*fragilis desf.*
**Alismataceae**		
	Alisma	*plantago – acquatica l.*
**Liliaceae**		
	Asphodelus	*fistolosus l.*
	Asphodelus	*microcarpus salzm.*
	Allium	*roseum l.*
	Allium	*neapolitanum cyr.*
	Allium	*palleas l. ssp. siciliense stearn*
	Allium	*nigrum l.*
	Scilla	*autumnalis l.*
	Scilla	*sicula tia.*
	Ornithogalum	*narbonense l.*
	Leopoldia	*comosa parl.*
	Asparagus	*albus l.*
	Asparagus	*acutifolius l.*
	Asparagus	*aphyllus l.*
	Asparagus	*stipularis*
**Amaryllidaceae**		
	Sternbergia	*lutea*
**Agavaceae**		
	Agave	*americana l.*
**Iridaceae**		
	Crocus	*longiflorus rafin*
**Orchidaceae**		
	Ophrys	*lutea cav.*
	Ophrys	*fusca link*
**Salicaceae**		
	Populus	*alba l.*
	Populus	*nigra l.*
	Salix	*alba l.*
**Juglandaceae**		
	Junglans	*regia l.*
**Fagaceae**		
	Castanea	*sativa miller*
	Quercus	*ilex l.*
	Quercus	*suber l.*
**Ulmaceae**		
	Celtis	*australis l.*
**Moraceae**		
	Morus	*alba l.*
	Ficus	*carica l.*
**Urticaceae**		
	Urtica	*dioica l*
	Urtica	*rupestris guss.*
	Urtica	*urens l.*

**Chenopodium**		
	Beta	*vulgaris l.*
	Chenopodium	*album l.*
**Portulacaceae**		
	Portulaca	*oleracea l.*
**Caryophyllaceae**		
	Stellaria	*media (l.) vill.*
	Silene	*fuscata link*
**Lauraceae**		
	Laurus	*nobilis l.*
**Papaveraceae**		
	Papaver	*rhoeas l.*
**Brassicaceae**		
	Cardaria	*draba desv.*
	Sisymbrium	*irio l.*
	Sinapsis	*arvensis l.*
	Sinapsis	*alba l.*
	Diplotaxis	*crassifolia dc.*
	Diplotaxis	*crucoides dc.*
	Brassica	*inaca ten.*
	Brassica	*nigra*
**Capparidaceae**		
	Capparis	*spinosa l.*
**Rosaceae**		
	Pyrus	*pyrasyer*
	Pyrus	*amygdaliformis vill.*
	Malus	*sylvestris miller*
	Mespilus	*germanica (l.)*
	Crataegus	*azalorus l.*
	Rubus	*ulmifolius schott*
	Fragaria	*vesca l.*
	Sanguisorba	*minor scop.*
	Rosa	*canina l.*
	Rosa	*sempervireas l.*
	Prunus	*avium l.*
	Prunus	*spinosa l.*
**Fabaceae**		
	Cercis	*siliquastrum l.*
	Ceratyonia	*siliqua l.*
	Anagyris	*foelida l.*
	Lupinus	*angustifolius*
	Lupinus	*albus l.*
	Onomis	*natrix l. ssp. ramosissima*
	Trigonella	*maritime l.*
	Trigonella	*corniculata l.*
	Medicago	*lupulina l.*
	Medicago	*sativa l.*
	Medicago	*scutellata miller*
	Medicago	*rugosa*
	Medicago	*marina l.*
	Medicago	*arabica hudson*
	Melilotus	*italic lam.*
	Melilotus	*indica all.*
	Melilotus	*infesta guss.*

	Trifolium	*strictum l.*
	Trifolium	*repens l. ssp.*
	Trifolium	*nigrescens viv. ssp.*
	Trifolium	*pratense l.*
	Robinia	*pseudoacacia l.*
	Astragalus	*boeticus l.*
	Vicia	*villosa roth*
	Vicia	*sativa l.*
	Lathyrus	*odoratus l.*
	Pisum	*sativum l.*
**Oxalidaceae**		
	Oxalis	*acetosella l.*
	Oxalis	*pes – caprae l.*
**Simaroubaceae**		
	Alianthus	*altissima (miller) swingle*
**Euphorbiaceae**		
	Euphorbia	*peplis l.*
**Anacardiaceae**		
	Pistacia	*lentiscus l.*
	Rhus	*coriaria l.*
**Vitaceae**		
	Vitis	*vinifera l.*
**Malvaceae**		
	Marva	*sylvestris l.*
**Cactaceae**		
	Opuntia	*ficus indica miller*
**Punicaceae**		
	Punica	*granatum l.*
**Myrtaceae**		
	Myrtus	*communis l.*
	Eucalyptus	*sp. pl.*
**Araliaceae**		
	Hedera	*helix l.*
**Apiaceae**		
	Eryngium	*campestre l.*
	Daucus	*carota l.*
**Plumbaginaceae**		
	Plumbago	*europea l.*
**Oleaceae**		
	Olea	*europea l. sylvestris*
	Olea	*europea l. europaea*
**Convolvulaceae**		
	Convolvulus	*siculus l.*
	Convolvulus	*arvensis l.*
**Boraginaceae**		
	Borago	*officinalis l.*
	Cerinthe	*major l.*
**Verbenaceae**		
	Verbena	*officinalis l.*
**Lamiaceae**		
	Rosmarinus	*officinalis l.*
	Sideritis	*romana l.*
	Salvia	*viridis*
	Origanum	*vulgare l.*

	Thymus	*capitatus l.*
	Mentha	*longifolia hudson*
**Solanaceae**		
	Solanum	*nigrum l.*
	Datura	*stramonium l.*

Table S5A: Qualitative Melissopalynological analysis for Greece (EL), Italy (IT), and Egypt (EG)

**Relative pollen frequencies (%)**		GREECE	EL_FIELD			hw = honey wax								ITALY		CH-IT-SI			m = market		HC = honeycomb												
**FAMILY**	**GENUS/SPECIES type**			hw		hw		hw		hw			hw		m	m	m	m	m			HC	HC	HC	HC	HC	HC	m	m	m	m	m	m
**NECTARIFEROUS PLANTS (N)**		1_1	2_1	2_2	3_1	3_2	4_1	4_2	5_1	5_2	6_1	7_1	7_2	1	2	3	4	5	6	7	8	9	10	11	12	13	14	15	16	17	18	19	20
Acanthaceae	Acanthus																											<1					
Alliaceae	Allium				1				2	<1	6																						
Anacardiaceae	Rhus																														1		
Apiaceae	Ferula-Scandix											2		<1	1				5							<1		6	6	17	8		[illegible]
Apiaceae	Daucus-Crithmum-Foeniculum-Carum														1													1					
Apiaceae	Smyrnium																															2	
Apiaceae	Tordyllium-Coriandrum-Anethum	<1	7	47	<1	2	<1		<1		1												11										
Araliaceae	Hedera										6	3													1	<1							
Asparagaceae	Asparagus/Other						1	7						2		1									1		9		5	<1			
Asphodelaceae	Asphodelus	<1			<1					<1							<1															<1	
Asteraceae	Anthemis	2			8		15	2	4	1	1		13	1	1			<1	1		2	3		<1	1		2	<1		<1	2		[illegible]
Asteraceae	Carthamus																										<1						
Asteraceae	Centaurea redempta/raphanina T													<1																			
Asteraceae	Centaurea solstitialis T																<1					2	2		9	14	1	1	<1				
Asteraceae	Cirsium	<1	2		2		1	1	2		<1	2		5	8	2	<1	2	18	2	2	3	<1	<1	4	12	<1	6	11	1	3	<1	
Asteraceae	Dittrichia/Inula	2															<1						2		<1								[illegible]
Asteraceae	Helianthus																																[illegible]
Asteraceae	Onopordum		2																														
Asteraceae	Taraxacum	2	<1	4	3			<1		2	1	11	2	1		1		1	<1			2	2		1	2		<1	<1				[illegible]
Boraginaceae	Anchusa																																
Boraginaceae	Borago														<1	<1	<1		<1			2	<1	<1				1	<1	<1	1	1	
Boraginaceae	Cynoglossum/Cerinthe															2		1				15	2	<1								1	
Boraginaceae	Echium	7		4	3	33	2	2			24	4		2	27	6	10	8	23			6	3					3	1	2	4		[illegible]
Boraginaceae	Echium italicum T								46																			1			1		
Boraginaceae	Lithospermum/Omphalodes													1	6	3	34		3	2									4	<1	1		
Boraginaceae	Myosotis																					3											
Brassicaceae	Draba, Eruca		2	7																			17			1							
Brassicaceae	Brassica/Sinapis	40	17		1	1	9	8	8	13	18	24	9	9	6	24	6	33	3	14	16	5	3	2	9	21	14	18	4	2	12	3	[illegible]
Brassicaceae	Lepidium sativum																																
Cactaceae	Opuntia														<1										<1								
Caesalpiniaceae	Ceratonia															2																	
Campanulaceae			<1												1																		[illegible]
Caprifoliaceae	Lonicera								<1	<1																							

Convolvulaceae	Convolvulus		1	<1	<1			<1		<1	<1				<1	<1	<1		<1	<1						<1		<1	<1	<1			
Cucurbitaceae	Ecballium/Bryonia						<1																										
Cucurbitaceae	Citrullus																																[illegible]
Cucurbitaceae	Cucurbita																																[illegible]
Dipsacaceae	Knautia	<1								<1	1	<1	<1																				
Dipsacaceae	Scabiosa																					1				<1	<1	<1	<1	<1	<1		
Ericaceae	Arbutus							<1					2																				
Ericaceae	Erica	2		<1	1					4	3		2																				[illegible]
Euphorbiaceae	Croton																																
Euphorbiaceae	Euphorbia																										1	<1		<1			
Fabaceae	Acacia															<1	<1	1			<1	<1						<1	<1	<1	<1	<1	
Fabaceae	Delonix regia																																
Fabaceae	Lotus	3												45	22	21	38	41	5	65	68	27	33	69	20	15	66	20	31		20		
Fabaceae	Medicago																	1										<1					
Fabaceae	Melilotus													3	8	2			3							1		<1	14		6	84	
Fabaceae	Ononis																<1												4	1	3	5	
Fabaceae	Ononis pubescens											2																					
Fabaceae	Robinia																			2													
Fabaceae	Trifolium incarnatum T																														3		[illegible]
Fabaceae	Trifolium pratens T									<1				<1	1				3			3	2	1				2	<1			1	
Fabaceae	Trifolium repens T	3								6				19	<1	<1	2	5	3			2		13	39	19			4	34	1	2	
Fabaceae	Vicia	<1	1	<1			1			2	3			<1		2			3	2									<1	24		<1	
Fagaceae	Castanea			2			3					7							10						1								
Geraniaceae															<1																<1		
Hyacinthaceae	Drimia		<1																														
Hyacinthaceae	Muscari											<1													4	<1							
Lamiaceae	Ballota														4	1									1						1		
Lamiaceae	Mentha																						2		3	1		<1		<1	1		
Lamiaceae	Ocinum																																
Lamiaceae	Phlomis/Lamium	<1	<1			1		1			<1	7																					
Lamiaceae	Rosemarinus				1						3	<1		<1	<1	1	<1											2	<1		1		
Lamiaceae	Stachys																									1							
Lamiaceae	Teucrium						<1	2									1									<1					1		
Lamiaceae	Thymbra/Thymus						1															8	2										
Liliaceae		2	<1		<1		1							1	<1		1		3	2	6	1						<1			1		[illegible]
Lythraceae	Lythrum																					8		2									
Malvaceae	Abutilon								<1																								

Malvaceae	Bombax																																
Malvaceae		<1	<1		<1	<1	<1	<1	<1		<1	<1	<1		<1	<1	<1	<1						<1		<1		<1	<1	<1	<1	<1	[illegible]
Malvaceae	Sida																																
Meliaceae	Melia																<1		3														
Musaceae	Musa																																
Myrtaceae	Eucalyptus			2						1	6			1		1							2					24		6			[illegible]
Myrtaceae	Myrtus/Psidium																						2	7	1	1					19		
Oleaceae	Ligustrum											2																					
Oxalidaceae	Oxalis				<1												<1	<1	<1					1				<1		<1	1	<1	
Pedaliaceae	Sesamum																																[illegible]
Polygonaceae	Fagopyrum																																[illegible]
Portulacaceae	Portulaca																																
Punicaceae (acc.Dimopoulos)	Punica																																
Ranunculaceae	Clematis		<1						<1		6							3				3	11	<1				1	1		1		
Ranunculaceae	Nigella																																
Rhamnaceae	Rhamnus	3															<1						3										
Rosaceae	Pyrus-Prunus	17	<1									1					<1	1			2			2	<1	<1		2		4	1		[illegible]
Rosaceae	Rubus									2				1	2	1	<1		<1	6	2	3	2		4	7	2	2			1	1	
**Rutaceae**	**Citrus**	17	66	35	79	63	66	75	36	67	24	33	73	9	10	31	4	3	18	4	2	3	2	2	<1	<1	5	3	12	1	5	1	
Salicaceae	Salix						1			2							<1														1		
Styracaceae	Styrax																							<1						<1			
Tamaricaceae	Tamarix																											1	2	5			[illegible]
Verbenaceae	Vitex																																[illegible]
	SUM	100	100	100	100	100	100	100	100	100	100	100	100	100	100	100	100	100	100	100	100	100	100	100	100	100	100	100	100	100	100	100	[illegible]
**NECTARLESS PLANTS (NL)**																																	
Anacardiaceae	Pistacia	2	1	<1	<1	1		<1	2	<1	2	9	1			1	<1		3	3	3	1				3	3	<1	12	4	<1	<1	
Arecaceae (Palmae)		1															<1																
Asteraceae	Artemisia																																[illegible]
Asteraceae	Xanthium																											<1					[illegible]
Betulaceae																																	
Caryophyllaceae		<1								<1			<1												<1	<1		<1					
Chenopodiaceae/Amaranthaceae		1			<1	<1	1	4			<1			3	2	1	<1				1	3			<1	1	1	<1	3				[illegible]
Cistaceae		<1	<1				1		<1	<1	<1	1		<1			<1	<1	<1	6	6						2	1			1	1	[illegible]
Cupressaceae						<1		<1	<1	<1	<1			1	<1	1					1					<1			<1			<1	
Cyperaceae								<1																									

Ephedraceae	Ephedra																				1						1						
Euphorbiaceae	Mercurialis					<1																											
Fabaceae	Calicotome-Genista								<1													4								1	1		
Fagaceae	Quercus coccifera T	68	13	10	67	22	69	31	22	41	54	50	37			5	<1	1	5	6		21					5	8	3	3	7	1	10
Fagaceae	Quercus ithaburensis T						<1									1			15	<1							1	2			2		5
Fumariaceae	Fumaria																											<1	<1		1		
Fumariaceae	Hypecoum														3	5	1	1		1					3	2	1						
Hypericaceae	Hypericum										<1																		1				
Juglandaceae	Juglans				<1		<1	<1	<1		1	<1																			1		
Moraceae	Morus						<1	<1	<1																								
Oleaceae	Fraxinus																													17			
Oleaceae	Olea	14	74	73	18	42	12	44	57	35	28	22	46	<1	3	13	1	3	5	3	17	2	2	3	<1	1	9	11	8	1	8		3
Papaveraceae		1	<1	3	<1	1	<1	<1	4	1	1	2		12	4	10	3	3	11	4	4	5	38	8	7	9	1	5	5	<1	4	2	
Pinaceae		<1	<1	<1				<1	<1	<1	<1	<1		<1		<1	<1	<1	<1	<1	1		<1		<1	<1		<1	<1	<1		<1	
Plantaginaceae	Plantago			<1					<1	<1	<1	1				1	<1								<1	<1	1	<1			<1		1
Platanaceae	Platanus																												1	<1			
Poaceae (Graminae)		<1	<1	<1		<1	<1				<1	<1		<1			<1	<1	<1		1	<1	<1	1	<1	<1		<1			<1	<1	
Poaceae (Graminae)	Zea																																1
Polygonaceae	Rumex						1				<1																	<1					
Primulaceae	Cyclamen		<1																														
Ranunculaceae	Adonis														5	1	<1					1		1	2	3							
Ranunculaceae	Anemone coronaria T																							<1									
Ranunculaceae	Anemone hortensis T									<1																							
Ranunculaceae	Ranunculus											<1					<1							1									
Scrophulariaceae	Verbascum	3	1						1	<1							1									1							
Solanaceae																																	1
Thymelaeaceae	Thymelaeae																																
Vitaceae	Vitis																			6					2	1	2					1	
Zygophyllaceae	Tribulus							<1			<1	<1																					
**%NECTARLESS (%NL)**	**SUM**	91	90	87	86	68	85	80	88	78	87	84	83	15	17	37	10	8	39	29	36	37	40	14	15	21	27	29	32	25	25	5	34

Table S5B: Quantitative Melissopalynological Analysis for Greece (EL), Italy (IT), and Egypt (EG)

	GREECE	EL_FIELD										
Sample No	1_1	2_1	2_2	3_1	3_2	4_1	4_2	5_1	5_2	6_1	7_1	7_2
PG/10 gr honey	27100	62600	68000	359200	28500	49100	28500	63600	15400	37500	18800	54900
PG nectariferous/10 gr honey	2500	6300	8600	51400	9000	7600	5500	7800	3400	4900	2900	9100

ITALY	CH_IT_SI																			
Sample No	1	2	3	4	5	6	7	8	9	10	11	12	13	14	15	16	17	18	19	20
PG/10 gr honey	4600	6700	10900	29600	11700	6200	4600	5200	6300	6400	29500	109900	32400	9300	16300	3900	45000	10000	10700	101500
PG nectariferous/10 gr honey	3900	5500	6800	26700	10700	3800	3300	3300	3900	3900	25400	93200	25500	6800	11500	2700	33700	7500	10100	66500

EGYPT	(Citrus, Clover, AMPs, Basil)							
Sample No	1	2	3	4	5	6	7	8
PG/10 gr honey	20500	128700	92800	26500	3600000	135400	246900	2024000
PG nectariferous/10 gr honey	14000	96700	87800	23600	2289200	115900	245600	729700

Table S6: Parameters from OPLS-DA analysis for each comparison, using the ropls R Bioconductor package. For the analysis the standard scaling method was used after log10 data transformation

Greece *vs* Italy -Negative ion mode

	R2X	R2X(cum)	R2Y	R2Y(cum)	Q2	Q2(cum)	Signif.
p1	0.1740	0.174	0.6220	0.622	0.5190	0.519	R1
o1	0.3600	0.533	0.2240	0.224	0.1970	0.197	R1
o2	0.1020	0.635	0.1010	0.325	0.0605	0.258	R1
o3	0.0814	0.716	0.0290	0.354	0.0504	0.308	R1
o4	0.0287	0.745	0.0165	0.371	0.0112	0.319	R1

R2X(cum) R2Y(cum) Q2(cum) RMSEE pre ort pR2Y pQ2
Total 0.745 0.992 0.838 0.0476 1 4 0.005 0.005

Greece *vs* Egypt -Negative ion mode

	R2X	R2X(cum)	R2Y	R2Y(cum)	Q2	Q2(cum)	Signif.
p1	0.1700	0.170	0.505	0.505	0.3890	0.389	R1
o1	0.4800	0.650	0.3290	0.329	0.3280	0.328	R1
o2	0.0853	0.735	0.1130	0.442	0.0579	0.385	R1
o3	0.0540	0.789	0.0328	0.475	0.0462	0.432	R1
o4	0.0246	0.814	0.0158	0.491	0.0520	0.484	R1

R2X(cum) R2Y(cum) Q2(cum) RMSEE pre ort pR2Y pQ2
Total 0.814 0.996 0.873 0.0352 1 4 0.005 0.005

Italy *vs* Egypt -Negative ion mode

	R2X	R2X(cum)	R2Y	R2Y(cum)	Q2	Q2(cum)	Signif.
p1	0.1290	0.129	0.7320	0.7320	0.5340	0.534	R1
o1	0.3610	0.490	0.0982	0.0982	0.1990	0.199	R1
o2	0.0800	0.570	0.1200	0.2180	0.0501	0.249	R1
o3	0.0922	0.662	0.0225	0.2410	0.0402	0.289	R1
o4	0.0293	0.692	0.0215	0.2620	0.0277	0.317	R1

R2X(cum) R2Y(cum) Q2(cum) RMSEE pre ort pR2Y pQ2
Total 0.692 0.994 0.851 0.0384 1 4 0.005 0.005

Greece *vs* Italy -Positive ion mode

	R2X	R2X(cum)	R2Y	R2Y(cum)	Q2	Q2(cum)	Signif.
p1	0.1490	0.149	0.3980	0.398	0.3320	0.332	R1
o1	0.4000	0.548	0.3470	0.347	0.2560	0.256	R1
o2	0.1010	0.649	0.1500	0.496	0.1110	0.367	R1
o3	0.0575	0.707	0.0601	0.556	0.0525	0.420	R1
o4	0.0400	0.747	0.0296	0.586	0.0188	0.439	R1
o5	0.0279	0.774	0.0101	0.596	0.0208	0.460	R1

R2X(cum) R2Y(cum) Q2(cum) RMSEE pre ort pR2Y pQ2
Total 0.774 0.994 0.792 0.0437 1 5 0.005 0.005

Italy *vs* Egypt -Positive ion mode

	R2X	R2X(cum)	R2Y	R2Y(cum)	Q2	Q2(cum)	Signif.
p1	0.0928	0.0928	0.5250	0.525	0.3060	0.306	R1
o1	0.4070	0.5000	0.2530	0.253	0.2990	0.299	R1
o2	0.0697	0.5700	0.1390	0.392	-0.0225	0.276	NS
o3	0.0614	0.6310	0.0554	0.448	0.0568	0.333	R1
o4	0.0448	0.6760	0.0195	0.467	0.0260	0.359	R1

R2X(cum) R2Y(cum) Q2(cum) RMSEE pre ort pR2Y pQ2
Total 0.676 0.992 0.665 0.0465 1 4 0.005 0.005

References

[1] Gazala, N. and Nowar, E., 2014. Survey of different pollen sources gathering by honey bee at Qunatir Al-khiria, Qaluobia Governorate. *Journal of Plant Protection and Pathology*, *5*(6), pp.755-771.

[2] Hassanien, Mai M., Microscopic and chemical analysis of honey and bee bread at certain apiaries in Qalyubia Governorate and available honey in local market, Egypt."*MSc thesis, Ain Shams University, Egypt* 2018
